# An APE1 inhibitor reveals critical roles of the redox function of APE1 in KSHV replication and pathogenic phenotypes

**DOI:** 10.1371/journal.ppat.1006289

**Published:** 2017-04-05

**Authors:** Canrong Zhong, Mengyang Xu, Yan Wang, Jun Xu, Yan Yuan

**Affiliations:** 1 Institute of Human Virology, Zhongshan School of Medicine, Sun Yat-Sen University, Guangzhou, Guangdong, China; 2 Key Laboratory of Tropical Disease Control, Ministry of Education, Sun Yat-Sen University, Guangzhou, Guangdong, China; 3 Research Center for Drug Discovery, School of Pharmaceutical Sciences, Sun Yat-Sen University, Guangzhou, Guangdong, China; 4 Guanghua School of Stomatology, Sun Yat-Sen University, Guangzhou, Guangdong, China; 5 Department of Microbiology, University of Pennsylvania School of Dental Medicine, Philadelphia, Pennsylvania, United States of America; Louisiana State University Health Sciences Center, UNITED STATES

## Abstract

APE1 is a multifunctional protein with a DNA base excision repair function in its C-terminal domain and a redox activity in its N-terminal domain. The redox function of APE1 converts certain transcription factors from inactive oxidized to active reduced forms. Given that among the APE1-regulated transcription factors many are critical for KSHV replication and pathogenesis, we investigated whether inhibition of APE1 redox function blocks KSHV replication and Kaposi’s sarcoma (KS) phenotypes. With an shRNA-mediated silencing approach and a known APE-1 redox inhibitor, we demonstrated that APE1 redox function is indeed required for KSHV replication as well as KSHV-induced angiogenesis, validating APE1 as a therapeutic target for KSHV-associated diseases. A ligand-based virtual screening yielded a small molecular compound, C10, which is proven to bind to APE1. C10 exhibits low cytotoxicity but efficiently inhibits KSHV lytic replication (EC_50_ of 0.16 μM and selective index of 165) and KSHV-mediated pathogenic phenotypes including cytokine production, angiogenesis and cell invasion, demonstrating its potential to become an effective drug for treatment of KS.

## Author summary

As a major AIDS-associated malignancy, Kaposi’s sarcoma (KS) is caused by Kaposi’s sarcoma-associated herpesvirus (KSHV). Currently there is no definitive cure for KS. In this study, we identified a cellular protein, namely APE1, as an effective therapeutic target for blocking KSHV replication and inhibiting the development of KS phenotypes. We showed that the redox function of APE1 is absolutely required for KSHV replication, virally induced cytokine secretion and angiogenesis. Blockade of APE1 expression or inhibition of APE1 redox activity led to inhibition of KSHV replication and reduction of cytokine release and angiogenesis. Furthermore, we identified a novel small molecular compound, C10, which exhibited specific inhibitory activity on APE1 redox function and was demonstrated to efficiently inhibit KSHV replication and paracrine-mediated KS phenotypes such as angiogenesis and cell invasion. As a potent inhibitor of APE1 redox, C10 not only has value in development of a novel therapeutics for KS, but also may be used in therapies for other human diseases such as leukemia, pancreatic cancer and macular degeneration.

## Introduction

Kaposi’s sarcoma-associated herpesvirus (KSHV), also termed human herpesvirus type 8 (HHV8), is a member of the γ-herpesviridae subfamily. This virus has been proven to be the etiological agent of Kaposi’s sarcoma (KS) [1]. Almost 100% of KS lesions, regardless of their source or clinic subtype (i.e., classic, AIDS-associated, African endemic, and post transplant KS), are infected with KSHV. KS is the most common malignancy associated with HIV-infection. About 20% of AIDS patients develop KS with most of them (60%) manifesting with oral lesions [2]. Additionally, KSHV is also associated with two lymphoproliferative diseases, namely primary effusion lymphoma (PEL) [3] and multicentric Castleman’s disease (MCD) [4]. Currently there is no definitive cure for KS and other KSHV-associate diseases.

The KSHV life cycle consists of two phases, latent and lytic [5]. When KSHV infects a target cell, it establishes latent infection by default and expresses a few latent genes to maintain latent infection. Establishment of latency is a viral strategy to avoid host immune surveillance and fuse symbiotically with the host for persistent infection. Spontaneous lytic replication occurs in a small portion (1–2%) of infected cells, releasing infectious virions to infect fresh cells in order to sustain the population of latently infected cells, that otherwise would be quickly lost by segregation of latent viral episomes as spindle cells divide [6]. Furthermore, KSHV lytic replication is also crucial for efficient dissemination from its long-term reservoir to the sites of disease and providing paracrine regulation for KS development [7–10]. Therefore, both latent and lytic cycles of KSHV are important for viral pathogenicity.

KSHV lytic replication may serve as a therapeutic target for treatment of KS due to its etiological role in KS. Currently classic cancer therapies are generally used to treat KS patients, which include surgical excision and radiation therapy for patients with a few lesions in a limited area and chemotherapy for patients with extensive or recurrent KS [2]. The chemotherapeutics that have been approved by the FDA include liposomal anthracycline products [11] (liposomal doxorubicin or liposomal daunorubicin) [12], paclitaxel and interferon-alpha [13–15]. However, these therapeutic agents do not target the etiological virus and the tumor response to any chemotherapeutic regimen is only transient. For AIDS-KS, HAART regimens are associated with regression in the size and number of existing KS lesions[16–19]. However, despite its dramatic decrease in frequency since the advent of HAART, KS remains the most common AIDS-associated cancer. In addition, there is an emergence of a new HAART-associated syndrome. In a subset of HIV-seropositive individuals, starting HAART in the setting of advanced HIV infection results in a paradoxical clinical worsening of the existing infection or the appearance of a new condition including KS in a process known as immune reconstitution inflammatory syndrome or IRIS[20,21]. Since IRIS-KS is the result of responses by a recovered immune system to KS-causing pathogen (i.e. KSHV), the treatment of KSHV-seropositive, HIV-positive patients with a combination of antiretroviral (HAART) and anti-KSHV chemotherapeutics is expected to yield positive results. However, there is currently no available drug effectively targeting KSHV.

Apurinic/apyrimidinic endonuclease 1 (APE1), also termed Redox factor-1 (Ref-1), is a multifunctional protein. Its C-domain carries a DNA base excision function (acting as apurinic/apyrimidinic endonuclease) and its N-domain has a redox activity controlling gene expression for cell survival pathways. The redox function of APE1 converts its substrate proteins from oxidized inactive form to reduced active form and affected transcription factors include AP-1 [22], NF-κB [23], Egr-1 [24], HIF-1α [25], p53 [26], Pax protein [27] and COX-2 [28]. The DNA base excision repair function of APE1 has been extensively explored as a therapeutic and chemopreventive target [29]. It was demonstrated that the redox function of this protein is also associated with many malignancies, such as pancreatic cancer [30], ovarian cancer [31], and glioblastoma [32]. Indeed the inhibition of APE1 redox function decreases cell proliferation, prevents the angiogenesis progress, and blocks the differentiation of endothelial precursor cells [33]. As many of the transcription factors under the regulation of the APE1 redox function are known to be involved in KSHV lytic replication and other pathogenic phenotypes such as neo-angiogenesis, endothelial cell differentiation and cell invasion, we asked if APE1 redox function is critically required for KSHV lytic replication and tumorigenesis and if the enzyme can serve as an effective target for drug development for KSHV-associated diseases. In this study, we have validated APE1 as an effective target for blocking KSHV lytic replication and some pathogenic phenotypes. A small molecular compound was identified to be a novel inhibitor of APE1 redox function through a ligand-based virtual screening using an in-house three-dimensional (3D) molecular superimposing algorithm, WEGA and shown to be effective in inhibition of KSHV lytic replication, virus-mediated endothelial differentiation, neo-angiogenesis and cell invasion.

## Results

### APE1 is essential for the lytic replication of KSHV

**Fig 1:**
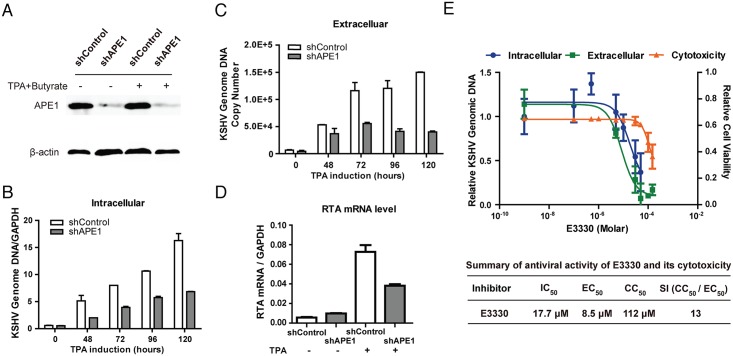
Validation of the role of APE1 redox function in KSHV DNA replication and virion production using shRNA-mediated silencing of APE1 expression and a specific APE1 inhibitor. (A) An APE1 shRNA lentivirus targeting the 3’UTR of APE1 (shAPE1), along with a non-targeting control shRNA lentivirus (shControl), were transduced into BCBL-1 cells. The effects of the shRNA on APE1 and β-actin gene expression were determined by Western blot. (B) The cells stably expressing APE1 and control shRNAs were induced by TPA for lytic viral replication. KSHV lytic DNA replication was evaluated at different time points up to 120 hour post-induction by real-time PCR with specific primers detecting ORF73 and GAPDH sequences. (C) KSHV virion production was assessed by determining encapsidated KSHV genomic DNA in the media 5 days after induction. (D) The RTA mRNA levels were also determined using qRT-PCR at 48 hour post-induction. (E) An APE1 redox inhibitor E3330 was examined for the effect on KSHV DNA replication, virion production and its associated cytotoxicity. The half maximal inhibitory concentration (IC_50_) of E3330 for KSHV DNA replication, the half maximal effective concentration (EC_50_) for blocking KSHV virion production and the half maximal cytotoxic concentration (EC_50_) were determined and selectivity index (SI)) was calculated as the ratio of CC_50_/EC_50_. The mean value of each datum was obtained from three independent experiments and presented with standard deviation. Statistical analysis was performed using t test, P-value was calculated by GraphPad Prism.

During the switch from latency to lytic replication, KSHV uses several signaling pathways to activate the viral Replication Transcription Activator (RTA) to initiate viral lytic replication cascade. It was reported that the MEK/ERK, JNK and P38 multiple mitogen-activated protein kinase pathways are necessary and sufficient for activating the RTA promoter through activation of the transcription factor AP-1 [34,35]. The DNA binding activity of AP-1 to its target DNA was found to be dependent on the redox activity of APE1 [22], suggesting that APE1 might be a regulatory factor for KSHV lytic replication. To explore this hypothesis, we first determined the role of APE1 in KSHV lytic replication through shRNA-mediated knock down of APE1 expression in cells and examining the effects of APE1 silencing on KSHV lytic replication. An shRNA against human APE1 was introduced into BCBL-1 cells by lentiviral transduction. In the transduced cells, the APE1 shRNA effectively down-regulated APE1 expression by 80% in comparison to the cells transduced with control shRNA (Fig 1A). Then the cells were treated with TPA to induce KSHV reactivation. The viral DNA replication was examined in these shRNA-transduced cells by measuring intracellular viral genomic DNA at different time points up to 120 hour post-induction using real-time PCR. The effect of APE1 knock down on progeny virion production was also determined. The culture media of shRNA-transduced cells were collected up to 5 days post-induction and the amounts of virion particles in the media were determined by quantifying encapsidated viral DNA with real-time PCR. Results showed that with APE1 knock down, both KSHV lytic DNA replication and virion production were significantly reduced (Fig 1B and 1C). The expression of immediate-early gene RTA in shRNA-transduced BCBL-1 was determined by qRT-PCR and the result showed a significant reduction of RTA transcript with APE1 knock down (Fig 1D).

The importance of the redox function of APE1 to KSHV lytic replication was further examined using a pharmacological inhibitor, namely E3330, which has been proven to be a redox function inhibitor of APE1 [36]. BCBL-1 cells were treated with TPA to induce lytic viral replication. Three hours after the induction, the cells were exposed to E3330 of various doses. Forty-eight hours post-induction, total DNA was extracted and intracellular viral genomic DNA was determined by real-time PCR. The half maximal inhibitory concentration of E3330 (IC_50_ for viral DNA replication) was calculated to be 17.7 μM (Fig 1E). The effect of E3330 on progeny virion production was also determined. Five days post-induction, the E3330-treated cell culture media were collected and virion particles were determined by quantifying encapsidated viral DNA in the media. The half maximal antiviral effective concentration (EC_50_) was calculated from extracellular virion doses response curves to be 8.5 μM (Fig 1E). Cytotoxicity of E3330 on BCBL-1 cells was assessed by Trypan blue exclusion that provided a homogeneous method for estimating both the numbers of viable and nonviable cells present in culture of each treatment. The half maximal cytotoxic concentration (CC_50_) was determined to be 112 μM, yielding a selectivity index (CC_50_ / EC_50_) of 13 (Fig 1E). Taken together, both studies with shRNA knock down and the pharmacological inhibitor validates that APE1 is essential for KSHV lytic replication and can serve as an effective target for antiviral agents against KSHV infection.

### APE1 is required for KSHV-induced angiogenesis of mesenchymal stem cells

**Fig 2:**
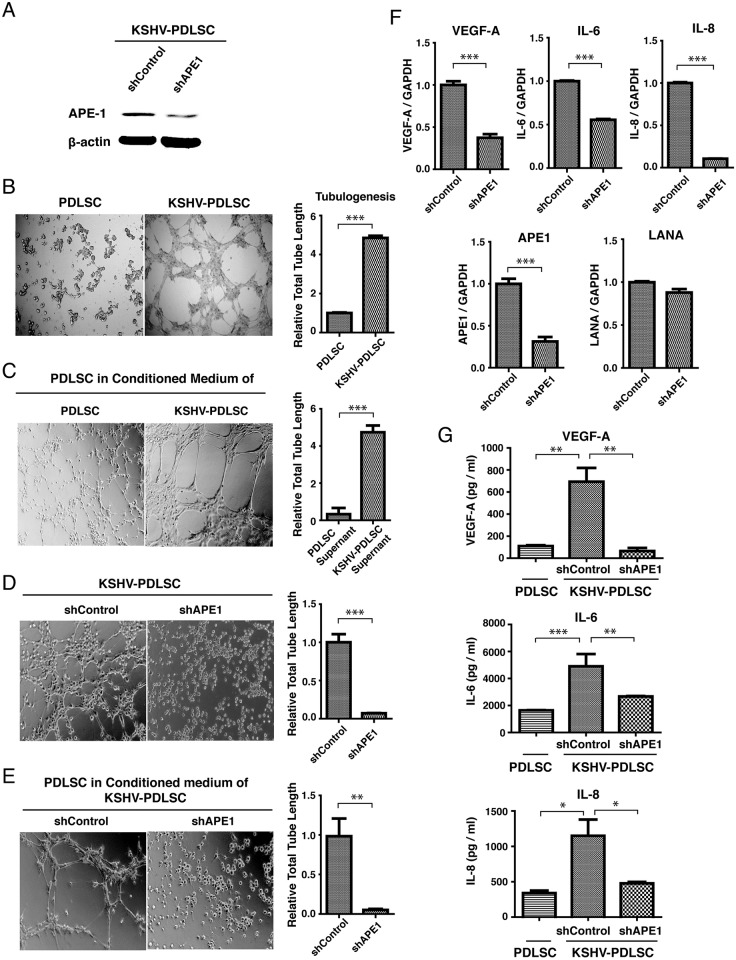
Critical role of APE1 in KSHV-mediated cytokine production and paracrine-regulated endothelial differentiation and angiogenesis. (A) To examine the role of APE1 on angiogenesis of PDLSCs, APE1 shRNA transduced cells were infected with KSHV and expression of APE1 were compared with that of control cells by Western bolting. (B) PDLSCs were infected by KSHV and applied on Matrigel to examine the ability for tubule formation. (C) Uninfected cells growing in conditioned medium of KSHV-infected PDLSCs were subjected for Tubule formation assay. (D) Capillary tube formation activities of KSHV-PDLSCs with shAPE1 and shControl were evaluated by tubule formation assay. Photographs were taken at 8h post-seeding. (E) Uninfected PDLSCs were treated with conditioned medium of shRNA-expressing KSHV-PDLSCs and the effect of APE1 knock down on paracrine-regulated angiogenesis was also examined by tubule formation assay. The degree of tubulogenesis was quantified by measuring total tube length and shown on the right of each panels. The effects of APE1 knock down on levels of VEGF-A, IL-6, IL-8 mRNAs as well as secretion of these cytokines to the culture media was determined by qRT-PCR (F) and ELISA (G), respectively. Statistical analyses were performed using two-paired t test or one-way ANOVA, followed by comparisons performed using Bonferroni method. P-value was calculated by GraphPad Prism. *P<0.05, **P<0.01 and ***P<0.001.

KS is an angiogenic and invasive tumor and abnormal neovascular channel that fills with red blood cells is a pathological feature of KS. Although the nature and cellular origin of KS cells remains contentious, KSHV infection of both endothelial cells and mesenchymal stem cells (MSCs) confer the cells with certain KS features including angiogenic, invasive and transformation phenotypes [37,38]. When human MSCs from periodontal ligament (PDLSC) were infected with KSHV, increased angiogenesis activity was shown in an *in vitro* Matrigel tubulogenesis assay (Fig 2B). In addition, KSHV-mediated angiogenesis has been demonstrated to involve paracrine regulation, and the leading hypothesis for the mechanism is that KSHV proteins, including LANA and vIRF-3, interact with and up-regulate HIF-1α, leading to increased VEGF-A expression [39–42]. KSHV induction of VEGF-A is responsible for the induction of angiogenesis, as well as proliferation of KS spindle cells. In addition, IL-6, IL-8, and other cytokines also play important roles in KSHV-associated angiogenesis. IL-6 and IL-8 are regulated by MAPK pathway through the transcription factor AP-1 in KSHV-infected cells [43,44]. We found that the conditioned media of KSHV-infected PDLSC could increase the angiogenesis activity of PDLSCs, indicating that KSHV can induce angiogenesis of oral MSCs in a paracrine manner(Fig 2C). Since both AP-1 and HIF-1α are known to be substrates of APE1 [45] and the redox activity of APE1 is reported to be associated with angiogenesis in cancer [28], we asked whether APE1 is crucial for KSHV-induced angiogenesis in PDLSCs. To this end, APE1 shRNA-transduced PDLSCs were infected with GFP-carrying recombinant KSHV (rKSHV.219) at an MOI (multiplicity of infection) of 20 (viral genome equivalent) and infection rates were from 72 to 87% on the basis of GFP expression. The effect of APE1 silencing on angiogenesis capacity of MSCs was assessed through a Matrigel tubulogenesis assay. The KSHV-PDLSCs transduced with control shRNA were able to form capillary-like tubules on Matrigel that represents the later stage of angiogenesis. By contrast, the KSHV-PDLSCs expressing APE1 shRNA showed reduced tubulogenesis activity (Fig 2D). Furthermore, paracrine-induced angiogenesis was also APE1-dependent as the conditioned medium of APE1 shRNA expressing cells showed lower capability to induce paracrine-associated angiogenesis (Fig 2E).

To confirm that APE1 is indeed required for KSHV-induced angiogenesis of PDLSCs through regulating the transcription and secretion of angiogenic growth factors, we examined the effect of APE1 silencing on the transcription and production of VEGF-A, IL-6 and IL-8 of KSHV-infected PDLSCs. Total RNAs were extracted from KSHV-PDLSCs transduced with control or APE1 shRNAs and subjected to quantitative real-time PCR using probes for VEGF-A, IL-6 and IL-8. The levels of APE1 and LANA mRNAs were also evaluated as references. The results showed that along with reduced expression of APE1, the levels of VEGF-A, IL-6 and IL-8 mRNAs were significantly decreased in APE1 knock down cells (Fig 2F). In parallel, the culture media of KSHV-PDLSCs with APE1 or control shRNA were collected and the levels of secreted VEGF-A, IL-6 and IL-8 were measured by ELISA. Results demonstrated dramatic reduction of these angiogenic factors in the culture media of APE1 shRNA-expression cells (Fig 2G). Therefore, we conclude that APE1 is a critical regulator for paracrine regulation of angiogenic growth of KSHV-infected KS progenitor cells.

### Screening for a potential inhibitor of the redox activity of APE1

**Fig 3:**
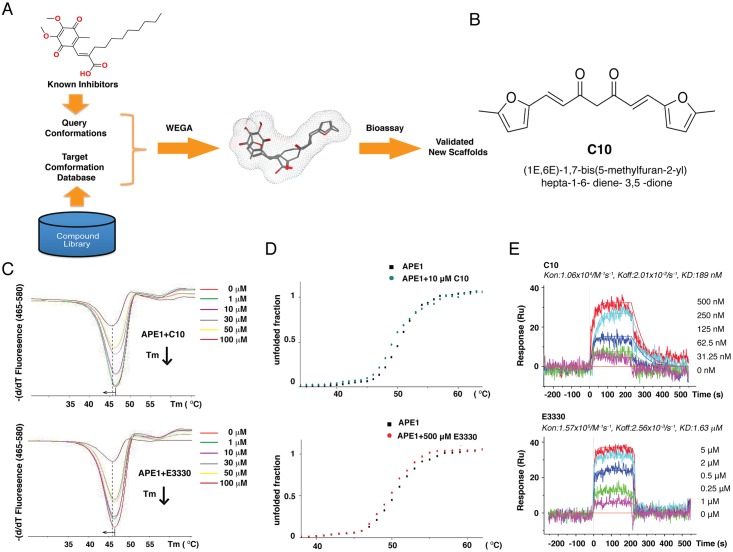
A ligand-base virtual screen led to identification of C10 as a potential APE1 inhibitor. (A) Schematic diagram of ligand-based virtual screen using a 3D molecular superimposing algorithm (WEGA) against a small molecular tangible library. (B) Structure of C10. (C) Differential scanning fluorimetry (DSF) assays were performed with various concentrations of either C10 and E3330 and the melting curves of APE1 were shifted to the left by 1.3°C and 1.0°C, respectively. (D) The effect of C10 and E3330 on APE1 melting curve as measured by circular dichroism (CD). The Melting curves of APE1 were shifted to the left with both C10 and E3330. (E) Surface plasmon resonance (SPR) was used to determine the binding constants of interaction C10 and E3330 with APE1 and the K_D_ values were shown.

APE1 is an important regulator of AP-1, HIF-1α, and many other transcription factors, of which many are known to be involved in KSHV replication and pathogenic processes. Thus, APE1 represents a promising target for antiviral against KSHV and associated diseases. In order to identify potential inhibitors against APE1, a ligand-based virtual screening was employed using an in-house three-dimensional (3D) molecular superimposing algorithm (WEGA). Using E3330 as a query molecule, compounds within our natural compound repository, Guangdong Small Molecule Tangible Library [46] were screened on the basis of 3D shape and pharmacophore features (Fig 3A). Conformational ensembles (maximum size of 250) were generated for each compound in the database through the CAESAR algorithm in Discovery Studio 3.5. Following shape-based alignment, the similarities between the query and target molecules were calculated by WEGA. Among the highly scored compounds exhibiting similar shape and pharmacophore features to E3330 were C10 (Fig 3B), which was chosen for further characterization.

### Interaction of C10 with APE1

Differential scanning fluorimetry (DSF) and circular dichroism (CD) were employed to test whether C10 is capable of interacting with APE1. DSF allows the identification of a compound binding to its target protein through the observed thermal shift (ΔTm) of the protein [47–49]. APE1 was incubated with C10 at 37°C for 30 min and then the fluorescence dye was added. The melting curves with increasing temperature were obtained from a real-time instrument LightCycler 480. Results showed that C10 shifted the Tm of APE1 by 1.3°C (Fig 3C), indicating the direct binding of C10 to APE1. As a reference, E3330 shifted the Tm of APE1 by 1.0°C. CD is an assay using polarized light to measure the melting temperature changes influenced by compound binding. The result also confirmed that C10 destabilizes the structure of APE1 and so does E3330 (Fig 3D).

The binding affinities of C10 and E3330 to APE1 were determined by using surface plasmon resonance (SPR) and Kd’s of interaction of C10 and E3330 with APE1 were measured to be 189 nM and 1.63 μM, respectively, indicating a higher binding affinity of C10 to APE1 than that of E3330 (Fig 3E).

### C10 inhibits the redox function, but not the DNA repair function of APE1

**Fig 4:**
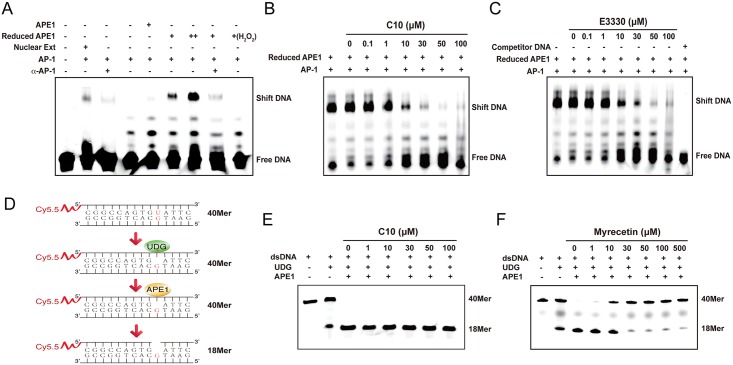
C10 inhibits the redox activity but not base excision repair function of APE1. (A) An EMSA-based assay for APE1 redox activity was established with purified AP-1 and APE-1 proteins. A double-stranded DNA carrying an AP-1 binding motif was mixed with either nuclear extract or purified c-jun/c-fos (1:1 ration). Purified APE1 (Supporting Information, S7 Fig) or reduced APE1 (treated with 0.25 mM DTT) were added to the reaction. AP-1-DNA binding was examined by EMSA. Nuclear extract and reduced APE1 resulted in shifted bands while oxidized APE1 (untreated and treated with H_2_O_2_) failed to support AP-1 DNA binding. Increasing concentrations of C10 (B) and E3330 (C) were added into the reaction and the effects of them on redox activities were measured through EMSA. (D) Schematic diagram of an *in vitro* assay for APE1 base excision function. A 40-bp double-stranded DNA with an U-G base pair reacted with UDG and APE1. After being denatured at 95°C, samples were dissolved on a 20% denaturing polyacrylamide gel. C10 (E) and Myrecin, a known APE1 DNA repair inhibitor (F), of different concentrations were added to the reaction and the effects of the compounds on the DNA repair activity of APE1 were measured.

Since the ability of AP-1 binding to its DNA binding sites has been shown to require redox regulation by APE1, electrophoresis mobility shift assay (EMSA) was employed to investigate the ability of C10 to inhibit the redox function of APE1. Although the double-stranded DNA fragment containing AP-1 binding motif could be shifted when incubated with a nuclear extract, purified AP-1 proteins (c-jun/c-fos heterodimer) failed to bind to the DNA fragment (Fig 4A). In accordance with redox regulation of APE1, the binding of AP-1 heterodimer to the double-stranded AP-1 oligonucleotides occurred only in the presence of reduced form of APE1 (Fig 4A). Untreated APE1 (oxidized form) showed almost undetectable AP-1 DNA binding activity, confirming that AP-1 DNA binding activity is dependent on redox function of APE1 in this system. To test whether C10 can inhibit APE1 redox activity and, as a consequence, block AP-1 DNA binding activity, the EMSA experiment was performed with increasing concentrations of C10. As shown in Fig 4B, a dose-dependent inhibitory response by C10 was established, yielding an IC_50_ of 17.0 μM. By comparison, a relatively weaker effect on APE1-dependent AP-1 binding was observed for E3330 (IC_50_ = 36.0 μM, Fig 4C).

To examine whether C10 also affects the C-terminal function of APE1, an *in vitro* assay for APE1 DNA excision activity was established. A 40-bp DNA with an U-G base-pair was prepared by annealing of two synthetic oligonucleotides. Uracil-DNA glycosylase (UDG) recognizes the U-G base pair and removes uracil base to product a single base lesion. APE1 is able to recognize the absent site and its exonuclease activity shortens the 40-bp DNA to 18 bp (Fig 4D). As shown in Fig 4E, the APE1 exonuclease activity was unaffected in the presence of up to 100 μM of C10. By contrast, Myrecetin, a known APE1 exonuclease function inhibitor and served as a positive control, showed a dose-dependent inhibitory response (Fig 4F). Taken together, the results demonstrated that C10 is an inhibitor specific to the APE1 redox function.

### C10 inhibits KSHV lytic replication through blocking AP-1 signaling and RTA expression

**Fig 5:**
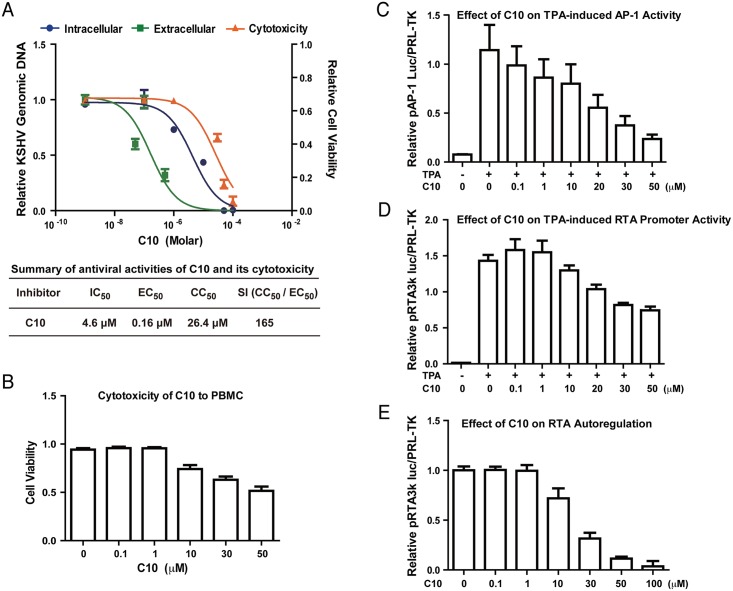
Effect of C10 on KSHV lytic replication. (A) BCBL-1 cells were treated with a wide range of concentrations of C10 three hours after being induced by TPA for viral lytic replication. Intracellular KSHV DNA replication (blue), extracellular virion production (green), and cell viability (orange) were determined as described in Materials and Methods. The mean values from three independent experiments and standard deviations are presented on the y-axis of dose-response curves. Calculated IC50, EC50 and CC50 are shown in the table. (B) The cytotoxicity of C10 to peripheral blood mononuclear cells (PBMC) was assessed after 48 hour treatment by trypan blue staining. 293T cells were transfected with AP-1 promoter-luciferase (C) or RTA promoter-luciferase reporter plasmids (D), followed by induction with TPA. C10 were added 6 hours post-induction. The promoter activities were measured after twenty-four hours. (E) 293T cells were also co-transfected with RTA promoter-luciferase reporter and RTA expression vector. C10 were added 6 hours post-transfection and luciferase assays were performed after twenty-four hours.

As KSHV lytic replication is dependent on the redox function of APE1, we wondered if the new APE1 inhibitor C10 effectively inhibits KSHV lytic replication. BCBL-1 cells were induced into lytic replication by TPA. Three hour post-induction, the cells were exposed to C10 in a wide range of concentrations. Intracellular viral genomic DNA was determined at 48 hour post-induction. Encapsidated viral DNA was measured for released virions in the media 5 days post-induction. The IC_50_ and EC_50_ values of C10 were determined from the dose-response curves of the intracellular DNA and extracellular virion to be 4.6 and 0.16 μM, respectively (Fig 5A). The cytotoxity of C10 was assessed for BCBL-1 using trypan blue exclusion method for cell viability at 48h and CC_50_ values was determined to be 26.4 μM, leading to a selectivity index (SI = CC_50_/EC_50_) of 165. In addition, the cytotoxicity of C10 to primary lymphocytes was also assessed with human peripheral blood mononuclear cells (PBMCs) after 48 hour treatment and data were shown in Fig 5B. The cytotoxicities of C10 to different types of cells, including PDLSC, iSLK.219, HUVEC and HEK293T, were also examined and shown in Supporting Information (S4 Fig).

KSHV lytic replication is controlled by the viral transcription activator RTA. Since RTA is known to be regulated by AP-1 signal transduction, inhibition of APE1 dependent AP-1 DNA binding would therefore disrupt this signaling. Thus, functional inhibition of APE1 redox activity by C10 is speculated to result in the decreased expression of RTA expression. To test this, we employed promoter-reporter assays to study the effects of C10 on the activation of the AP-1 promoter as well as the RTA promoter by AP-1 (c-Jun/c-fos). 293T cells were transfected with an AP-1 promoter-luciferase reporter plasmid or an RTA-promoter-luciferase reporter, respectively. Six hours late, C10 was added into the culture media in varying concentrations. TPA was then added 24h post-transfection to activate AP-1 signaling. The activation of the AP-1 promoter as well as the RTA promoter in the absence and presence of C10 were measured through the luciferase activities 36 hours post-induction. C10 was able to block TPA-induced AP-1 promoter (Fig 5C) and RTA promoter activities in a dose-dependent manner (Fig 5D). These results indicate that AP-1 redox inhibitor C10 blocks KSHV lytic replication through inhibiting TPA-induced AP-1 pathway and RTA expression.

Interestingly, C10 was also found to be able to block auto-regulation of the RTA promoter activity by RTA itself (Fig 5E). This result suggests that APE1 redox function is also required in some event downstream of RTA such as activation of RTA or associated cellular protein(s). To examine if C10 is able to block KSHV lytic replication induced by ectopically expressed RTA, we employed iSLK.219 cell line in that KSHV lytic replication is under control of doxycycline-inducible RTA [50,51]. iSLK.219 was treated with doxycycline (DOX) for 3 hours, then C10 in a wide range of concentration was added to the culture medium. In this system, C10 was found to be able to inhibit viral lytic DNA replication with an IC_50_ value of 5.8 μM and virion production with an EC_50_ value of 3.2 μM, respectively (Supporting Information, S1 Fig).

### C10 inhibits KSHV-induced angiogenesis of oral MSCs

**Fig 6:**
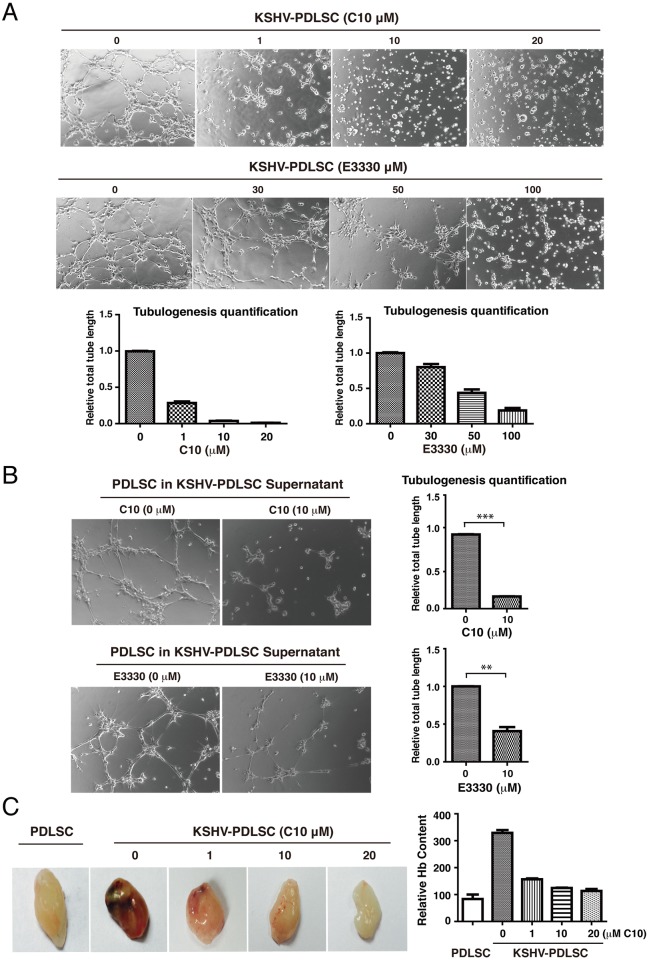
Effect of C10 on KSHV-mediated paracrine regulation of endothelial lineage differentiation and angiogenesis of MSCs. (A) KSHV-infected PDLSCs were placed on Matrigel in the presence of C10 and E3330 at different concentrations. Tubulogenesis was examined under a microscope and quantified by measuring the total tube length using the Image J software. (B) KSHV-PDLSCs were treated with C10 and E3330 for 24 hours. Medium was replaced with new one to remove the inhibitor compounds. Conditioned medium was collected after 24 hours. Effects of C10 and E3330 on tubulogenesis of PDLSCs treated with conditioned medium of KSHV-infected PDLSCs culture were analyzed on Matrigel. (C) Effect of C10 on angiogenesis of KSHV-PDLSCs *ex vivo* was examined using Matrigel plugs implanted into C57BL/6 mice. After 7 days, Matrigel plug were removed and photographed. After Matrigel plugs were homogenized and centrifuged, their supernatant was quantified for hemoglobin content using Drabkin's reagent (right panel). Statistical analyses were performed using t test, P-value was calculated by GraphPad Prism. *P<0.05, **P<0.01 and ***P<0.001.

KS is a vascular tumor and pathological neoangiogenesis is a hallmark of the cancer. As KSHV-mediated angiogenesis or angiogenic growth factor production is dependent on APE1 (Fig 2), possibly through maintenance of redox status of AP-1 and HIF-1α and their DNA binding activities, we investigated whether C10 could be an effective inhibitor of KSHV-elaborated neoangiogenesis. In a Matrigel tubulogenesis assay, the ability of KSHV-infected oral MSCs to form capillary-like tubules was examined after treatment with C10. As shown in Fig 6A, the tubulogenesis of KSHV-PDLSC was inhibited by C10 in a dose dependent manner. E3330 was also included in the assay as a reference and showed inhibitory activity at higher doses (Fig 6A). In addition, C10 and E3330 were capable of inhibiting paracrine-mediated angiogenesis with conditioned media from KSHV-infected PDLSCs (Fig 6B). We also examined C10 for its effect on the angiogenic capability of KSHV-infected HUVECs and their conditioned media. Similar inhibitory effects of C10 on angiogenesis of endothelial cells were observed in comparison to that of PDLSCs (Supporting Information, S2 Fig).

Next, we employed a murine Matrigel plug assay to examine the ability of C10 to inhibit angiogenesis *ex vivo*. KSHV-infected and mock-infected PDLSCs were mixed with Matrigel (without any cytokines) and implanted into C57BL/6 mice. In day 7, the matrigel plugs were stripped and examined for new blood vessel formation. As shown in Fig 6C, KSHV-infected PDLSCs exhibited increased blood vessel formation on the Matrigel plug. When C10 in varying concentrations was mixed with Matrigel, KSHV-induced angiogenesis was dramatically inhibited in a dose dependent manner (Fig 6C). The effect of C10 on KSHV-mediated angiogenesis in the *ex vivo* assay was quantitated by measuring the hemoglobin content after homogenizing the Matrigel plugs using Drabkin’s reagent [52]. The result, shown in Fig 6C (right panel), is consistent with that of *in vitro* Matrigel tubule formation assay.

### C10 blocks the production of angiogenic growth factors of KSHV-infected MSCs

**Fig 7:**
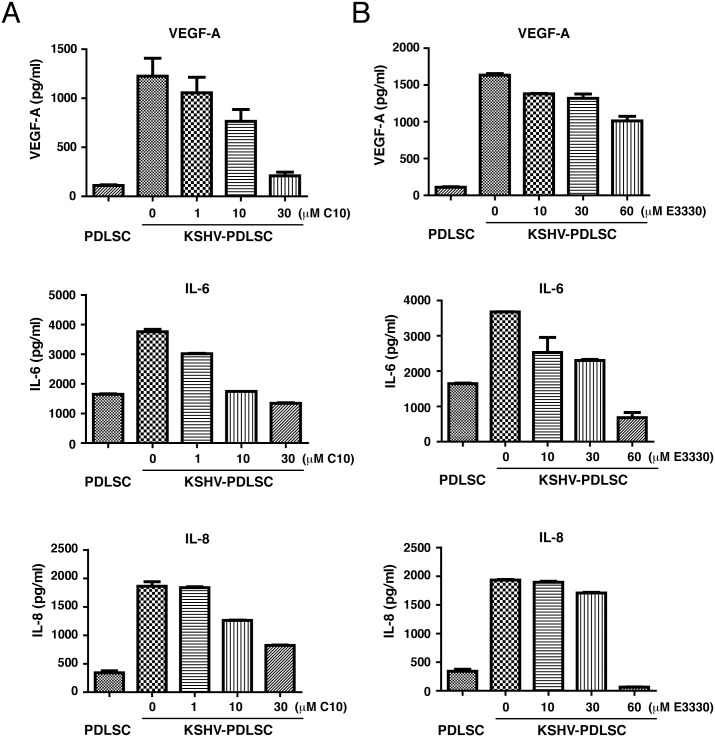
Effects of C10 and E3330 on KSHV-mediated secretion of VEGF-A, IL-6 and IL-8 from PDLSCs. KSHV-infected PDLSCs were treated with either C10 (A) or E3330 (B) for 24 hours. After changing media to remove C10 and E3330 from the media, cells were continued to culture for 24 hours. Then the culture media were collected and examined for the expression of VEGF-A, IL-6 and IL-8 by ELISA.

KS has been considered as a cytokine disease as KS lesions over-produce cytokines, especially angiogenic growth factors, that contribute to the major pathogenic features of KS [53,54]. In the early stage, KS is not a real sarcoma but an angiohyperplastic-inflammatory lesion mediated by inflammatory cytokines and angiogenic factors [55]. The finding that the paracrine-regulated angiogenesis requires the redox function of APE1 (Fig 2) prompted us to further investigate if blockade of APE1 redox function by C10 could reduce cytokine and angiogenic factor production. KSHV-infected MSCs were shown to produce a wide array of cytokines that resembles KS cells. KSHV-infected PDLSCs were treated with either C10 or E3330 for 24 hours. After changing media to remove C10 and E3330 from the media, cells were continued to culture for 24 hours. Then the culture media were collected and examined for the expression of VEGF-A and IL-6 by ELISA. The results showed that both C10 (Fig 7A) and E3330 (Fig 7B) were able to reduce the levels of these angiogenic growth factors. Similar results were also obtained with HUVECs where KSHV-induced productions of VEGF-A, IL-6 and IL-8 were inhibited by C10 (Supporting Information, S3 Fig).

### APE1 is required for cell migration and invasion of KSHV-infected MSCs and C10 is able to block the process

**Fig 8:**
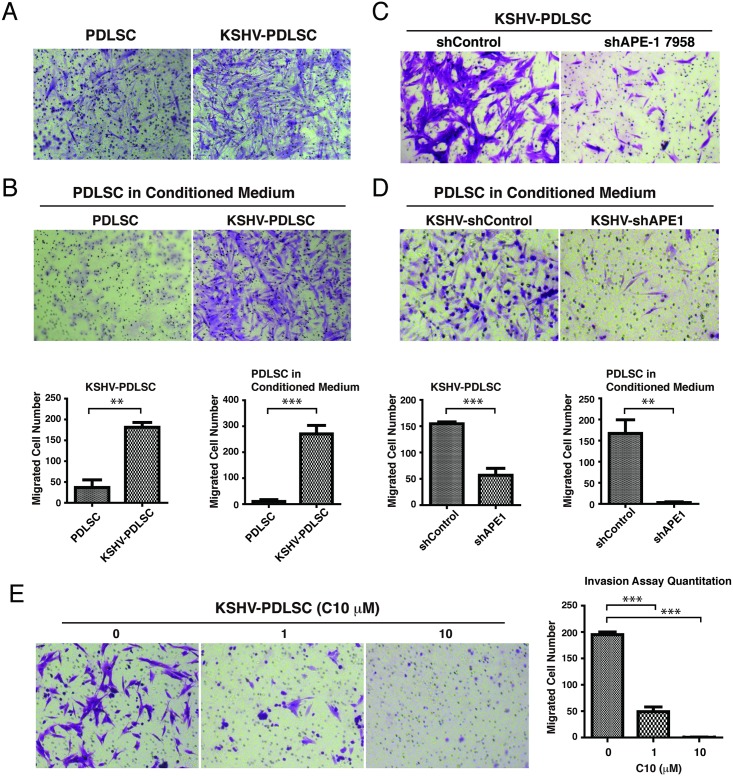
APE1 is required for KSHV-induced cell invasion and C10 inhibits this process. Transwell migration assay was employed to assess cell invasion ability. (A) PDLSCs or KSHV-PDLSCs (1.5x10^4^ cells / well) were seeded in the upper chamber of Transwell in serum-free a-MEM. a-MEM containing 10% FBS was used as a stimulus for chemotaxis in the lower chamber. Cells migrated to the lower chamber were stained with crystal violet. (B) PDLSCs were seeded in the upper chamber and conditioned media of PDLSCs or KSHV-PDLSCs were used as stimulus for chemotaxis in the lower chamber. (C) APE1 was knocked down using shRNA approach in KSHV-infected PDLSCs and the effects on cell invasion were measured by Transwell migration assay. (D) Conditioned medium of KSHV-PDLSCs in which APE1 has been silenced by shRNA was used as chemotaxis in Transwell migration assay. (E) KSHV-infected PDLSCs were treated with C10 in different concentrations and effect of C10 on cell invasion were assessed by Transwell migration assay. Quantification of transwell-invasion assay was performed by Image J software by counting from multiple randomly selected microscopic visual fields. Statistical analyses were performed using t test or one-way ANOVA test. P-value was calculated by GraphPad Prism. *P<0.05, **P<0.01 and ***P<0.001.

Cell invasion is a feature of KS and KSHV infection of human primary endothelial cells has been reported to promote cell migration and invasion [56,57]. Using a Matrigel-transwell assay, we investigated the invasive property of KSHV-infected PDLSCs. Cells were stimulated for chemostasis in a-MEM containing 10% FBS and seeded in a Matrigel on the upper chamber of a Transwell containing a-MEM free of FBS. The cells that migrated through the membrane were visualized by crystal violet staining. KSHV-infected PDLSC showed increased migration and invasion activity (Fig 8A). In addition, PDLSCs in the KSHV-PDLSC conditioned medium also had elevated migration and invasion (Fig 8B), suggesting the KSHV-mediated cell migration is paracrine regulated, possibly through VEGF [57]. shRNA-mediated knock down of APE1 led to a significant decrease in cell invasion ability in both KSHV-infected PDLSCs and the use of the conditioned media (Fig 8C and 8D). Consistently, C10 was also able to block cell migration and invasion of KSHV-PDLSC (Fig 8E). Taken together, APE1 is required for KSHV-mediated cell migration and invasion and APE1 redox inhibitor C10 can efficiently block this phenotype.

### The mechanism of C10 action as a small molecular inhibitor of APE1 redox function

**Fig 9:**
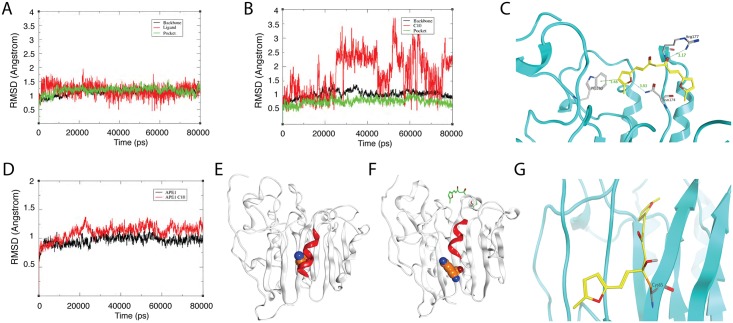
Molecular dynamics simulation-based Prediction of binding mode of C10 with APE1. (A) RMSD plots of C10 (red), the backbone atoms (black) and the C-terminal pocket (green) during MD simulation. (B) RMSD plots of C10 (red) in N-terminal region (green). (C) The binding mode of C10 with the C-terminal pocket. C10 binding sites are depicted in cyan ribbon and C10 in yellow stick with the surrounding residues in gray. (D) Comparison between RMSD backbone curves of APE1 (black) and C10-bound APE1 (red). (E) Full view of APE1 crystal structure in which Cys65 (orange) is buried by α-helix (red). (F) Structure of APE1 bound by C10 (green) in the C-terminal pocket. The C10 binding lifts up the α-helix (red) and expose Cys65 (orange) to the solvent. (G) Molecular modeling of C10 in the redox reactive site. C10 (yellow) makes a covalent bond with the S atom of Cys65 of APE1 (cyan).

C10 was identified through a ligand-based virtual screen using E3330 as a template molecule and proven to be a new inhibitor of APE1 redox function. C10 blocks the redox function of APE1 and shows no demonstrable effect on the base excision endonuclease activity (Fig 4). The two functions of APE1 are independent and separable in the primary amino acid sequence. The cysteine at position 65 (Cys65) is critical for the redox function [58]. However, APE1 crystal structure showed that Cys65 is deeply buried inside the protein [59,60], raising a question how C10 interacts with APE1 and gains access to the redox active site. In the early study on E3330, it was surprisingly found that E3330 binds at the C-terminal domain, distant from Cys65 in a NMR study [61]. At high concentration, E3330 interacts with both regions of the N-terminus (amino acids 68−74) and C-terminus (amino acids 266−273) as mapped by hydrogen/deuterium exchange mass spectrometry [36]. The results suggest that a locally unfold state could be the redox active state of APE1. To understand how C10 binds to APE1 and blocks redox function, molecular docking and molecular dynamics (MD) simulation were performed on the ligand-receptor complex system. First, the time-dependent root mean square deviation (RMSD) values of the complex backbone and ligand trajectories were acquired for 40 ns MD simulation. When C10 was docked at the C-terminal pocket, MD simulations produced stable RMSD curves, which fluctuated around 1.3 Å (Fig 9A), suggesting that C10 fits well in the C-terminal pocket. However, if C10 was docked into the N-terminal domain, the RMSD curves fluctuated 2 to 4Å (Fig 9B), indicating that C10 was easily dropped out from the N-terminal pocket. These data suggest that C10 prefers to bind with the C-terminal rather than the N-terminal domain of APE1. This is consistent with the crystal structure, which shows that the redox active site of APE1 is buried inside the protein. Then, we docked C10 into the C-terminal pocket and performed MD simulations to explore the binding pose of C10 in the pocket. The binding mode derived from MD simulation results indicated that C10’s aromatic groups prefer to form σ–π interactions with Trp280 and its carbonyl group trends to form hydrogen bonds with Arg177 side-chain (Fig 9C). Now the question is how C10, through interacting with those amino acids in the C-terminal, affects APE-1 redox activity. Toward this end, we compared the RMSD curves of APE1 and APE1-C-C10 backbone trajectories in 40 ns MD simulation. The RMSD curve for APE1 backbone was stable, while the RMSD curves for APE1-C-C10 backbone show higher fluctuations (ranging from 1 to 1.5 Å) in comparison with APE1 alone with RMSD within a range from 0.5 to 1Å (Fig 9D). This result informs that C10 binds with the C-terminal pocket and results in conformation transitions of APE-1. It is observed from the MD simulation that in the absence of C10, Cys65 is hidden behind an α-helix in APE1 (Fig 9E), while binding of C10 into the C-terminal pocket lifts up the α-helix domain, therefore resulting in Cys-65 exposed to the solvent (Fig 9F). This conformational change (or called locally unfolded) allows C10 to form a covalent bond with Cys65, as revealed in a covalent docking (Fig 9G), which leads to the blockage the formation of the disulfide bond between Cys-65 and Cys-95 and inhibition of the redox activity of APE1.

## Discussion

In the current study, we validated APE1 as an effective target for blocking KSHV replication and treating KS and other KSHV-associated malignancies. This study revealed crucial roles of APE1 in KSHV lytic replication, as well as in the development of KS pathogenic features such as cytokine production, angiogenesis and cell invasion. Furthermore, a novel compound was identified that binds to APE1 and blocks its redox function, thereby exhibiting antiviral activity against KSHV and KS pathogenic feature development. The salient features and implication of these findings are as follows.

(1) APE1 is a multifunctional protein with the DNA base excision activity in its C-terminal region and redox activity in its N-terminal domain. These two functions are completely independent in their actions as the mutation of Cys65 abolishes the redox function but does not affect the DNA repair function, whereas the mutation of His309, which is required for the DNA repair function, does not affect the redox function[62]. Both functions of APE1 have been intensively explored for therapeutic purposes. Selective inhibitors against APE1 base excision repair activity have been exploring to be used in combination with DNA-interactive anticancer drug to enhance the efficacy of chemotherapy [63]. The APE1 redox regulation of a class of transcription factors affecting cancer survival and growth makes the protein attractive to be used as a target for cancer therapeutic strategy [64]. APE1 is overexpressed in human pancreatic cancer. The specific APE1 redox inhibitor E3330 is reported to cause tumor growth inhibition in cell lines and pancreatic cancer xenograft model in mice, demonstrating the potential of APE1 inhibitors in pancreatic cancer treatment [30]. In the current study, we, for the first time, demonstrated that APE1 plays crucial roles in KSHV lytic replication as well as other critical aspects of KS development through its redox function and validated APE1 as an effective target for halting viral replication and treatment of KS. Among the transcription factors whose transcriptional activities are dependent on APE1 redox activity, many are known to participate in KSHV life cycle and pathogenicity. (i) KSHV reactivation is initiated through mitogen-activated protein kinase (MAPK)/extracellular signal-regulated kinase (ERK) pathway or Ras/Raf/MEK/ERK/Ets-1 pathway that lead to activation of RTA by AP-1[34,35,65]. Through redox regulation, APE1 determines if AP-1 is able to activate the RTA promoter. Therefore, APE1 redox function is absolutely required for KSHV reactivation and lytic replication. (ii) KS is considered a cytokine disease and KS cell growth and angiogenesis is dependent on autocrine and paracrine cytokine regulation. Pathogenic roles of HIF-1α, AP-1 and COX-2 in KSHV-mediated overexpression of a variety of cytokines have been established [35,41,66]. APE1 is known to regulate HIF-1a, AP-1 and COX-2 and, as a consequence, controlling cytokine production[22,25,28]. Our results are consistent with the previous reports and confirm the roles of APE1 in KSHV-induced cytokine production, validating APE1 as an effective target for halting KSHV replication and inhibiting development of KSHV-associated malignancies. The critical roles of APE1 in KSHV-associated cytokine production through redox regulation of DNA binding activities of the transcription factors including HIF-1α, COX-2 and AP-1 in turn explain the pharmacological mechanism underlying inhibition of KSHV-mediated cytokine production, angiogenesis and cell invasive growth by APE1 inhibitor C10.

(2) In addition to the well-characterized target proteins of APE1 mentioned above, our study suggests that APE1 may be involved in other steps in KSHV life cycle or pathogenicity by regulating other APE1 substrate proteins that have not yet been elucidated. (i) KSHV reactivation is regulated through a positive feedback mechanism [67]. RTA auto-regulates its own expression at the transcriptional level by using cellular RBP-Jκ notch signaling pathway[68,69]. We found that the APE1 redox function is required for RTA auto-regulation (Fig 5E), suggesting that some protein involving the auto-regulation, such as RBP-Jκ or RTA, might be a substrate of APE1 and under the redox regulation by APE1. (ii) The EC_50_ of C10 for blocking virion particle release is 29-fold lower than IC_50_ for inhibiting intracellular viral DNA replication (Fig 5A), suggesting APE1 has an additional role in a certain step between viral DNA replication and virion assembly (e.g. regulation of late gene expression), which can also be affected by C10. Further investigation is warranted for the roles of APE1 in these steps of viral life cycle, which may lead to identification of new substrates of APE1 and expand our understanding on the role of APE1 in biology.

(3) Since KSHV is heavily dependent on APE1-regulated cell proliferation and survival pathways for viral replication and pathogenic feature development, APE1 has been validated to be an excellent target for anti-KSHV and KS therapy. Its inhibitors are able to affect not one but many steps towards KSHV replication and KSHV-associated malignancy development. Multi-target drugs have raised considerable interest in the last decade due to their advantages in the treatment of complex diseases[70]. Although C10 is a specific inhibitor of APE1 and not an authentic multi-target compound in a narrow sense, it practically impacts multiple proteins and affects multiple biological processes. Therefore, C10 has a greater potential than a compound that affects a single biological reaction in becoming an effective drug to treat complex diseases such as KS and other cancers. However, the multiple targeting property of a compound often raises an issue of cytotoxicity. Therefore we have closely paid attention to the cytotoxicity issue with C10. In cell-based assays, C10 showed relatively low cytotoxicity with an acceptable selectivity index (e.g., SI = 165 for inhibition of KSHV virion production in B cells). E3330 was also reported to exhibit low cytotoxicity [71]. It could be explained by that the transcription factors under APE1 redox regulation are mainly over-expressed in malignant or virally infected cells so that APE1 redox inhibitors affect cancer cells and viral replication in a greater extent than normal somatic cells[72,73]. This notion is supported by the fact that APE1 redox function is not evolutionally conserved but only present in mammals. In contrast, the base excision repair function of APE1 is conserved during phylogeny, which is an ortholog of *E*. *coli* Xth (exonuclease III) [74]. Nevertheless, the safety and cytotoxicity of C10 is an important issue that warrants further evaluation in future pre-clinical and clinical research.

(4) APE1 is associated with the progression of various human diseases[62]. The redox function of the enzyme regulates several pathways relevant to cell proliferation and cancer survival [64]. Dysregulation of APE1 has been reported to be associated with cancer where alteration of APE1 was found in both expression and subcellular localization levels [30,32]. Inhibition of APE1 redox function decreases cell proliferation and migration of cancer cells, and blocks the differentiation of endothelial precursor cells and angiogenesis [33]. As a novel and effective inhibitor of APE1 redox function, C10 has potential to become an anti-cancer agent to treat cancers and other angiogenesis-related human diseases. In addition to cancer, APE1 was shown to be associated with non-malignant angiogenesis-related disease. Aged macular degeneration (AMD) is the leading cause of severe vision loss in the elderly and neovascularization in the retina is a key pathology in AMD and other ocular diseases[75]. Currently the most advanced treatment for AMD is to block retinal neoangiogenesis using anti-VEGF agents, but the responses to the therapy are usually transient and disease recurrence occurs[76]. Inhibition of APE1 redox function appears to be a better strategy as APE1 inhibitors with multi-targeting property not only blocks VEGF production, but also reduces inflammatory reaction that also contributes to AMD development. It has been shown that inhibition of APE1 redox activity by E3330 blocks retinal angiogenesis *in vitro* and in an animal model[77,78]. As an effective APE1 redox inhibitor, we see potential value of C10 in treatment of AMD and other angiogenesis-related diseases.

Taken together, revelation of the multiple-roles of APE-1 redox function in KSHV lytic replication and viral pathogenic feature development (cytokine production, angiogenesis and migration) and the discovery of a novel APE1 redox inhibitor inform a new strategy toward controlling KSHV infection and treating KSHV-associated diseases.

## Materials and methods

### Ethic statements

This research was approved by the Animal Ethics Review Board of Sun Yet-sen University and Medical Ethics Review Board of Sun Yet-sen University. We have an existing Institutional Animal Care and Use Committee approval for the animal use (Approval No. 2015–041, Animal Ethics Review Board of Sun Yet-sen University). The experiment was carried out strictly following the Guidance suggestion of caring laboratory animals, published by the Ministry of Science and Technology of the People's Republic of China. The human sample collection and the use of PBMCs and PDLSCs in our research were approved the Medical Ethics Review Board of Sun Yet-sen University (Approval No. 2015–028). Written informed consent was provided by study participants.

### Cell culture

BCBL-1 cells (obtained from the National Institutes of Health AIDS Research and Reference Reagent Program), an effusion lymphoma cell line that latently infected with KSHV, were grown in RPMI 1640 medium supplemented with 10% heat-inactivated fetal bovine serum (FBS) and penicillin-streptomycin (50 units/ml) and 100 mg/ml amphotericin B sodium deoxycholate. Peripheral blood mononuclear cells (PBMCs) were isolated from whole blood of a healthy donor (obtained from the Zhongshan School of Medicine, Sun Yat-Sen University) and cultured in RPMI1640 medium supplemented with 10% FBS and antibiotics. Human embryonic kidney HEK293T cells were purchased from American Type Culture Collection (ATCC) and cultured in Dulbecco's modified Eagle's medium (DMEM) supplemented with 10% FBS and antibiotics. Periodontal ligament stem cells (PDLSCs) (obtained from the Hospital of Stomatology of Sun Yat-sen University) were isolated from the periodontal ligament tissues (n = 5 referred to 5 independent cultures from different individuals) and maintained in alpha minimal essential medium (aMEM, GIBCO Life Technologies) containing 10% FBS, 200 mM L-glutamine and antibiotics.

### shRNA-mediated knock down of APE1 expression

An shRNA lentiviral vector targeting the 3’-UTR site of APE1 mRNA (Clone ID: NM_080649.1-1305s1c1) was purchased from Sigma-Aldrich. Lentiviral particles were prepared by transfection HEK293T cells with pLKO.1-shAPE17958 (or pLKO.1-shControl), psPAX2, pMD2.G plasmids in the ratio of 4:3:1. Media containing lentiviruses were harvested at 48 h and 72 h and used to transduce BCBL-1 cells and PDLSCs. Transduced BCBL-1 cells were selected under 2 μg/ml puromycin for a week, while KSHV-PDLSCs was transduced transiently and cells were used 72 h after transduction.

### Analysis of intracellular and extracellular KSHV genomic DNA content and chemical effects

BCBL-1 cells were treated with TPA to induce KSHV lytic replication. Three hour post-induction, APE1 inhibitors in a wide range of concentration were added into the culture medium. Total DNA was purified using a DNasey kit (Magen) 48h post-induction. KSHV genomic DNA copy number was quantified by real time PCR on a Roche LightCycler 480 instrument using primers specified for LANA and normalized to GAPDH. The half-maximal inhibitory concentration (IC_50_) values of compounds were determined from a dose-response curve of KSHV DNA content values from TPA-induced and chemical-treated cells. The viral DNA contents with those of uninduced cells subtracted were divided by those of the control cells with no drug treatment and then represented on the y-axes of dose-response curves: y-axis value = (TPA_X_ − no TPA_X_)/(TPA_0_ − no TPA_0_), where X is any concentration of the drug and 0 represents nondrug treatment. The IC_50_ on viral DNA synthesis for each compound was calculated using GraphPad Prism software.

Extracellular virion numbers were estimated by determining encapsidated viral genomic DNA. Five days postinduction with TPA, BCBL-1 culture media were collected and virion particles were cleared by passing through 0.45-μm filters. Virions were pelleted from the medium supernatant. The virion preparations were treated with Turbo DNase I (Takara) at 37°C for 1 hour followed by proteinase K digestion. Encapsidated viral DNA was extracted with phenol-chloroform. Extracted DNA was precipitated with ice-cold ethanol, and the final DNA pellet was dissolved in TE buffer. The KSHV genomic DNA in virions was measured by real-time PCR with primers directed to LANA (ORF 73) as described above. Virion DNA copy numbers were calculated from a standard curve established using rKSHV.219. KSHV virion numbers were presented as the copy numbers of viral genomic DNA per milliliter of culture supernatant.

### Cytotoxicity assay

BCBL-1 and PBMCs were treated with chemical inhibitors in a wide range of concentration for 2 and 5 days. The viability of cells was assessed by counting Trypan blue-stained cells using a Countstar instrument. The half-maximal cytotoxic concentration (CC_50_) was calculated from dose-response curves with Graph-Pad Prism software. The cytotoxicity of chemical inhibitors to cells was also assessed in cell metabolic level using MTT assay. PDLSCs were plated in 96-well plates at 5000 cells/well and treated with inhibitors in various concentrations for 48 h. The medium was replaced with DMSO. Absorbance of MTT was measured at 570nm.

### Synthesis of C10

The structure of C10 [(1E,6E)-1,7-bis(5-methylfuran-2-yl) hepta-1-6-diene-3,5–dione] was acquired from a tangible compound repository, the Guangdong Small Molecule Tangible Library [46]. The compound C10 used in this study was synthetic, prepared by using the procedure illustrated in Supporting Information (S5 Fig). Boric anhydride (0.42 g, 6 mmol) and acetylacetone (3.00 g, 30 mmol) were suspended in 10 mL EtOAc, and the mixture was stirred for 3 h at 70°C. After removing the solvent, the resultant solid was washed with hexane. Then 20 ml of EtOAc, 5-methylfuran-2-carbaldehyde (6.00 g, 54 mmol), and tributyl borate (1.38 g, 6 mmol) were added and the mixture was stirred for 24 h. Butylamine (21.90 mg, 0.3 mmol) dissolved in EtOAc was added and the mixture reacted at 70°C for 24 h. Then 1 N HCl was used to adjust the pH to 5. The product was extracted from the water layer with EtOAc. The C10 was purified by recrystalization from EtOAc to yield 3.75 g red crystal, giving a percent yield of 90. The purity of C10 was determined by HPLC equipped with ZORBAX SB-C18 column (4.6x250mm, 5μm particle size) and a UV/VIS detector setting of λ = 254nm and 460nm. Compound was eluted with CH_3_OH solvent system and assayed by HPLC, which confirmed the purity of the compound to be ≥99% (Supporting Information, S5B Fig). ^1^H-NMR and ^13^C-NMR spectra data were recorded on a Bruker AvanceIII spectrometer at 400 MHz using TMS as reference (Bruker Company, USA). Detailed results can be found in Supporting Information (S6 Fig).

### Expression and purification of APE1, c-jun and c-fos proteins in *E*.*coli*

APE1 protein was expressed and purified from *E*. *coli* and used to study the interaction between APE1 with C10. AP-1 proteins (c-jun and c-fos) were purified for APE1 enzymatic assays. APE1 and c-fos cDNAs were cloned into the pET-28a vector with a hexahistidine (6xHis) tagged at the N-terminus. c-jun was cloned into pGEX-4T-1 vector with a GST tag. The *Roseta E*.*coli* transformed with each of the plasmids, were grown in LB media and induced with 1mM isopropylb-D–thiogalactoside (IPTG) when the culture reached the density of 0.6 OD. The bacterial culture grew at 37°C for 4 h for APE1 and at 25°C for 4 h for c-jun and c-fos. Pelleted cells were resuspended and sonicated in lysis buffer containing PMSF with (for GST-tag) or without DTT (for His-tag). His-tagged APE1 and c-fos were purified with Ni^2+^-NTA-resin and eluted with buffer containing imidazole. GST-tagged c-jun was purified using glutathione beads and eluted with reduced glutathione. Protein concentrations were determined using the BCA protein assay kit (Thermo Scientific).

### Differential Scanning Fluorimetry (DSF)

Purified APE1 protein (Supporting Information, S7 Fig) was diluted to 2 μM and incubated with C10 or E3330 in different concentrations at 37°C for 30 min. Fluorescence dye (SYPRO orange, Invitrogen) was added to a final concentration of 5x to the protein solution. Scanning was performed in a Lightcycler 480 instrument and run the temperature scan from 25–95°C min^-1^. Melting curve was plotted by the software LightCycler 480 Software release 1.5.0 SP4 (Roche). Melting temperature (Tm) of each concentration was obtained by LightCycler 480 Protein Melting software (Roche).

### Circular Dichroism (CD)

Far-UV CD spectra were acquired on a Chirascan spectropolarimeter (Applied Photophysics Ltd., Leatherhead, UK) in a 225−260 nm interval. APE1 (5 μM) with C10 (10 μM) or E3330 (500 μM) were performed in in 10 mM phosphate buffer (pH 7.4), using a 0.1 cm path-length cuvette. Thermal denaturation profiles were obtained by measuring the temperature dependence of the signal at 230 nm in the range of 20−90°C with a resolution of 0.5°C and a 1.0 nm bandwidth. A temperature controller was used to set up the temperature of the sample; the heating rate was 1°C min^-1^. Data were collected at 0.2 nm resolution with a 20 nm/min scan speed and a 4s response and were reported as the unfolded fraction versus temperature.

### Surface Plasmon Resonance (SPR)

Interactions of APE1 with C10 and E3330 were analyzed by using a ProteOn XPR36 SPR instrument (Bio-Rad, Hercules, CA). APE1 protein in 20 nM of 10 mM sodium acetate buffer, pH 5.5 was immobilized using amine coupling on the EDAC/Sulfo-NHS-activated surface of GLH biosensor chip channel L1 (Bio-Rad). The surface was blocked with 1M ethanolamine. The final immobilization level for APE1 was approximately 12,000 RU. Channel L2 (reference channel) of GLH biosensor chip was also activated with EDAC/Sulfo-NHS and blocked with 1M ethanolamine. Compounds in phosphate buffered saline (PBS), pH 7.4, containing 0.005% Tween-20 (PBST), were injected at 20 ml/min for 240s at concentrations of 10–3.125×10^−4^ μM (1:2 dilutions). Following the compound injection, the chip surface was regenerated with 40s pulses of 0.85% H_3_PO_4_ and running buffer. All experiments were performed at 25°C. In each of the kinetic studies, the interactions of C10 in six concentrations with APE1 and reference channel (L2) were monitored in parallel. The compound concentration data collected were reference-subtracted using ProtedOn Manager 2.0. Each set of sensorgrams was globally analyzed using the 1:1 Langmuir binding model to obtain the kinetic rate constants (Kon and Koff). Global kinetic rate constants (k_a_ and k_d_) were derived for each reaction, and the equilibrium dissociation constant, K_D_, was calculated using the equation K_D_ = k_d_ / k_a_.

### Electrophoretic Mobility Shift Assay (EMSA) for APE1 redox activity

An EMSA-based assay was adapted to measure APE1 redox activity following the APE-1-dependent AP-1 DNA binding ability [36,79]. APE1 was reduced by incubating in 0.25 mM DTT over night at final concentration of 1 μM. Reduced APE1 (2 μL, the final concentration was 0.1 μM) were incubated with purified c-jun/c-fos (1:1 ratio) in EMSA reaction buffer (10 mM Tris-HCl pH 7.5, 50 mM NaCl, 5mM MgCl_2_) for 20 min at 37°C. cy5.5-labelled double-stranded DNA was added and incubated for another 20 min. The samples were resolved on 5% nondenaturing polyacrylamide gels (TBE-PAGE) at 4°C, 100V for 1 h and then scanned with an Odyssey imager (LI-COR). Antibodies against c-jun and c-fos (Abclone) were included in the EMSA for supershift of specific band.

### Exonuclease assay for APE-1 C-terminal activity

A 40-bp double-stranded DNA with an U-G mismatch pair was prepared by annealing the following two oligonucleotides: Cy5.5–5’-GTAAAACGACGGCCAGTGUATTCGAGCTCGGTACCCGGGG-3’ and 5’-CCCCGGGTACCGAGCTCGAATGCACTGGCCGTCGTTTTAC-3’. The substrate DNA was incubated with Uracil-DNA glycosylase (UDG) at 37°C for 10 min. Uracil-DNA glycosyase removes the uracil-base from the U-G base-pair to produce an abasic site. Then APE1 was added with its buffer and incubated for another 30 min at 37°C. APE1 recognizes the abasic-site and cleave the DNA at the abasic site leading to release of the cy5.5- fluorophore labeled 18-nucleotide fragment, which can be resolved from the uncleaved 40-nucleotide fragment (intact substrate) on 20% denaturing polyacrylamide gels (TBE-Urea PAGE) and visualized with an Odyssey imager (LI-COR).

### Reporter plasmids and luciferase assay

The promoter sequence of the RTA promoter (3kb) was cloned into pGL3-basic vector (Promega) to generate pRTA-luc. The promoter-reporter plasmids pAP-1-luc was provided by Dr. Ersheng Kuang at Sun Yat-sen University. Subconfluent 293T cells grown in 48-well plates were co-transfected with 50 ng of pAP-1-luc or pRTA-luc and 5ng of pRL-TK by using lipofectamine 2000 reagent (Life Technologies). Twenty-four hours after transfection, cells were treated with 12-O-Tetradecanoyl-phorbol-13-acetate (TPA). The pRL-TK plasmid expresses Renilla luciferase and was used as an internal control. For RTA auto-regulation assay, 293T cells were co-transfected with pRTA-luc, pRL-TK and pCR3.1-ORF50. Thirty-six hour post-induction, the luciferase assay was performed with Promega's Dual-luciferase assay kit. Each sample was duplicated and each experiment was repeated at least three times.

### KSHV preparation and infection

iSLK.219 cells (kindly provided by Ke Lan lab of Institut Pasteur of Shanghai, Chinese Academy of Sciences, Shanghai, China), carrying rKSHV.219 [51,80], were induced for lytic replication by 1 μg/ml doxycycline and 1 mM sodium butyrate for 5 days. The culture media were filtered through a 0.45-m filter and centrifuged at 100000 g for 1 h. The pellet was resuspended in 1/100 volume of 1X PBS and stored at -80°C until use. PDLSCs were seeded at 2x10^5^ cells per well in 6-well plates. Cells were infected with KSHV in the presence of polybrene (5 μg/ml) at an MOI = 20 (viral genome copy equivalent). After two hours, the inoculum was removed and replaced with fresh culture medium. Cells were incubated under 5% CO_2_ at 37°C.

### *In vitro* tube formation assay

Forty eight-well plates were coated with Matrigel (100μl/well) and incubated at 37°C for 1h to allow gelation to occur and avoid bubble. PDLSC or KSHV-PDLSC were resuspended in 200 μl a-MEM without FBS and placed on the top of the gel. The cells was incubated at 37°C with 5% CO2 for 8 h, and images of tube formation were captured using a ZEISS fluorescence microscope. The quantification of the tube was using the software ImageJ to measure the total length of tube in the image. The average value was used for the histogram.

### *Ex vivo* matrigel plug assay

Female C57BL/6 mice (4 to 6 weeks old) were obtained from the Sun Yat-sen University Animal Center. Five animals were used per treatment group. 5-10x10^6^ cells in 100–200 μl medium were prepared and thoroughly mixed with 500 μl matrigels to total volume of 600–800 μl, and subsequently implanted to mice by inguinal injection. After even days, the mice were killed and matrigel plugs were removed. The Matrigel plugs were photographed and subsequently dissolved in 1 ml Dispase reagent for 16 h at 37°C. After removal of debris by centrifugation, hemoglobin was qualified using Drabkin's reagent (Sigma-Aldrich).

### Cell invasion assay

Cell invasion assays were carried out in 24-well transwell units (millipore). Briefly, polycarbonate filters with 8-μm pores were coated with 60 μl of matrigel-gel. PDLSCs or KSHV-PDLSCs (15,000 cells) in serum-free media were placed in the upper wells and the lower chambers were filled with 10% FBS medium. After 24 h incubation the cells that had passed through the filter were stained with crystal violet. The number of migrated cells was counted from multiple randomly selected microscopic visual fields using ImageJ software. Photographs were obtained and independent experiments were performed in triplicate.

### Enzyme-Linked Immunosorbent Assay (ELISA)

Levels of VEGF-A, IL-6 and IL-8 in cell culture supernatants were determined using specific ELISA kits for human VEGF-A (eBioscience), IL-6 (BD) and IL-8 (Xin Bosheng, China) according to the manufacturer instructions.

### Molecular docking

Virtual molecular docking of C10 with APE1 protein was executed using Autodock suite 4.2.6 [81]. APE1 protein was downloaded from Protein Data Bank (PDBID: 4QHE). Structure files of ligands were prepared for molecular docking by defining the number of torsion angles, addition of hydrogen atoms and conversion into software specific file format (pdbqt). Similarly, APE1 protein was also prepared by removing bad contacts, addition of hydrogen atoms, removal of needless water molecules, and conversion of file format into pdbqt. 5 Å near residues 68−74 and 266−273 on APE1 were defined as E3330 binding site over APE1 [36,61]. First, a prepared ligand was virtually docked against APE1 protein blindly. Then, the ligand was docked at the defined binding site using Lamarckian Genetic Algorithm of Autodock 4.2.

### Molecular dynamics simulations

The GPU accelerated Amber Molecular Dynamics suite with Amber ff99SB force field was employed for the all atoms explicit MD simulations of receptor-ligand complexes (http://ambermd.org/#Amber12). The receptor-ligand complexes were solvated with TIP3P water model in a cubic periodic boundary box to generate required systems for MD simulations and systems were neutralized using appropriate number of counterions. The distance between box wall and the complex was set to greater than 10Å to avoid direct interaction with its own periodic image. Neutralized system was then minimized, heated up to 300 K temperature and cooled down to equilibrated until the pressure and energies of systems were stabilized. Finally, the equilibrated systems were used to run 40 ns MD simulations. The average structures were calculated from the equilibrated stage of the MD trajectories (from 35 to 40 ns) and subsequently optimized with steepest descents for 200 steps. The minimized average structure was then used for the covalent docking.

### Covalent docking

DOCKovalent [82] is a covalent adaptation of DOCK3.6 [83,84]. Given a pre-generated set of ligand conformation and a covalent attachment point, it exhaustively samples ligand conformations around the covalent bond and selects the lowest energy pose using a physics-based energy function. For the docking reported in this work, Cys65 residue was defined as attachment site. The bond angle (C-ligand covalent attachment point-rest of ligand) of 109.5 ± 10°, also in 2.5° increments. Scoring was as described [82] using a physics-based energy function which uses pre-calculated van der Waals electrostatics (calculated with DELPHI), and solvent-excluded de-solvation [83] grids. The receptor is kept fixed throughout the docking simulation.

### Statistical analyses

All data were analyzed by two-tailed Student’s t-test and one-way ANOVA in GraphPad Prism, followed by comparisons performed using Bonferroni method. p values < 0.05 were considered significant (*P<0.05, **P<0.01 and ***P<0.001).

## Supporting information

S1 Fig Effect of C10 on RTA-initiated KSHV lytic replication in iSLK.219 cells. iSLK.219 cells were treated with doxycycline (DOX) for 3 hours, then C10 in a wide range of concentration was added to the culture medium. Intracellular KSHV genomic DNA replication (blue) and extracellular virion production (green) were determined as described in Materials and Methods. The values were compared to those from the control cells (nondrug treatment). The mean values of results from three independent experiments and standard deviations are presented on the y-axis of dose-response curves. The calculated IC50, EC50 and CC50 were shown in the table.
(PDF)

S2 Fig Effect of C10 on KSHV-mediated paracrine regulation of angiogenesis of KSHV-infected HUVEC. (A) KSHV-infected HUVECs were placed on Matrigel in the presence of C10 in different concentrations. Tubulogenesis was examined under a microscope and quantified by measuring the total tube length using the Image J software. (B) KSHV-HUVEC were treated with C10 for 24hours, cells were washed twice with PBS, fresh medium was replaced to discard compounds. Conditioned medium was collected for another 24hours. Effects of C10 on tubulogenesis of HUVECs treated with conditioned medium of KSHV-infected HUVECs culture were analyzed on Matrigel. Equal numbers of HUVEC cells was seeding on matrigel in the conditioned medium produced by KSHV-HUVEC, C10 treated or not. Photographs were taken at 8h post-seeding.
(PDF)

S3 Fig Effects of C10 on KSHV-mediated secretion of VEGF-A, IL-6 and IL-8 from HUVECs. KSHV-infected HUVECs were treated with either C10 for 24 hours. After changing media to remove C10 from the media, cells were continued to culture for 24 hours. Then the culture media were collected and examined for the expression of VEGF-A, IL-6 and IL-8 by ELISA.
(PDF)

S4 Fig Cytotoxicities of C10 to PDLSC, iSLK.219, HUVEC and HEK293T cells were assessed after treatment with C10 of different concentrations as indicated for 48 hours using MTT assay. (PDF)

S5 Fig Synthesis of C10 and HPLC spectrum of the compound. (PDF)

S6 Fig (A) 1H-NMR spectrum of C10 in CDCl3 (400MHz) and (B) 13C-NMR spectrum of C10 in CDCl3 (400MHz). (PDF)

S7 Fig Purification and purity of APE1 protein used in the study. (A) His-tagged APE1 was expressed in *E*. *coli*, purified by Nickel affinity chromatography with Ni2+-NTA-resin and eluted with buffer containing imidazole. (B) HPLC spectrum of obtained APE1 to show purify of APE1 used in the DSF, CD, SPR and EMSA assays.
(PDF)
