# Supplementary material for: An APE1 inhibitor reveals critical roles of the redox function of APE1 in KSHV replication and pathogenic phenotypes
Source: PLoS Pathog. 2017 Apr 5;13(4):e1006289. doi: 10.1371/journal.ppat.1006289 (PMC5381946; doi:10.1371/journal.ppat.1006289)
Supplement: S1 Fig — iSLK.219 cells were treated with doxycycline (DOX) for 3 hours, then C10 in a wide range of concentration was added to the culture medium. Intracellular KSHV genomic DNA replication (blue) and extracellular virion production (green) were determined as described in Materials and Methods. The values were compared to those from the control cells (nondrug treatment). The mean values of results from three independent experiments and standard deviations are presented on the y-axis of dose-response curves. The calculated IC50, EC50 and CC50 were shown in the table. (PDF) [file ppat.1006289.s001.pdf]

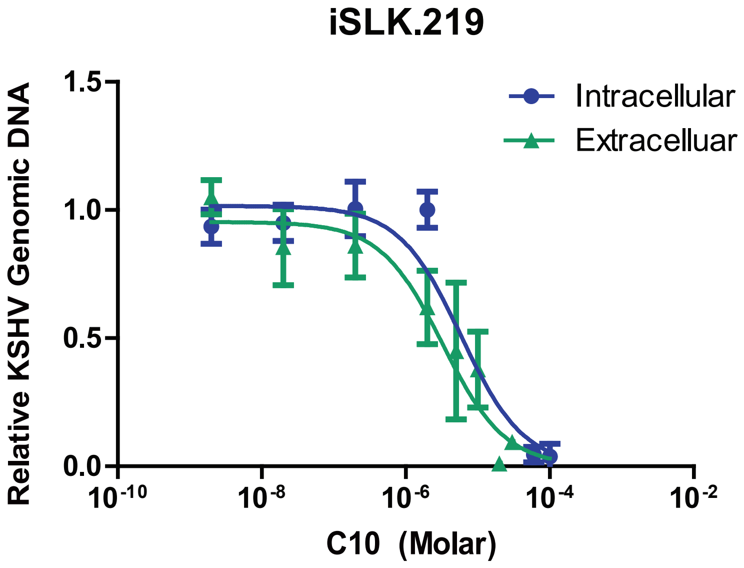

Summary of antiviral activities of C10 in iSLK.219

Inhibitor	IC_{50}	EC_{50}	CC_{50}	SI (CC_{50} / EC_{50})
C10	5.8 μM	3.2 μM	75 μM	23.4

Fig. S1. Effect of C10 on RTA-initiated KSHV lytic replication in iSLK.219 cells. iSLK.219 cells were treated with doxycycline (DOX) for 3 hours, then C10 in a wide range of concentration was added to the culture medium. Intracellular KSHV genomic DNA replication (blue) and extracellular virion production (green) were determined as described in Materials and Methods. The values were compared to those from the control cells (nondrug treatment). The mean values of results from three independent experiments and standard deviations are presented on the y-axis of dose-response curves. The calculated IC50, EC50 and CC50 were shown in the table.
