# Supplementary material for: An APE1 inhibitor reveals critical roles of the redox function of APE1 in KSHV replication and pathogenic phenotypes
Source: PLoS Pathog. 2017 Apr 5;13(4):e1006289. doi: 10.1371/journal.ppat.1006289 (PMC5381946; doi:10.1371/journal.ppat.1006289)
Supplement: S2 Fig — (A) KSHV-infected HUVECs were placed on Matrigel in the presence of C10 in different concentrations. Tubulogenesis was examined under a microscope and quantified by measuring the total tube length using the Image J software. (B) KSHV-HUVEC were treated with C10 for 24hours, cells were washed twice with PBS, fresh medium was replaced to discard compounds. Conditioned medium was collected for another 24hours. Effects of C10 on tubulogenesis of HUVECs treated with conditioned medium of KSHV-infected HUVECs culture were analyzed on Matrigel. Equal numbers of HUVEC cells was seeding on matrigel in the conditioned medium produced by KSHV-HUVEC, C10 treated or not. Photographs were taken at 8h post-seeding. (PDF) [file ppat.1006289.s002.pdf]

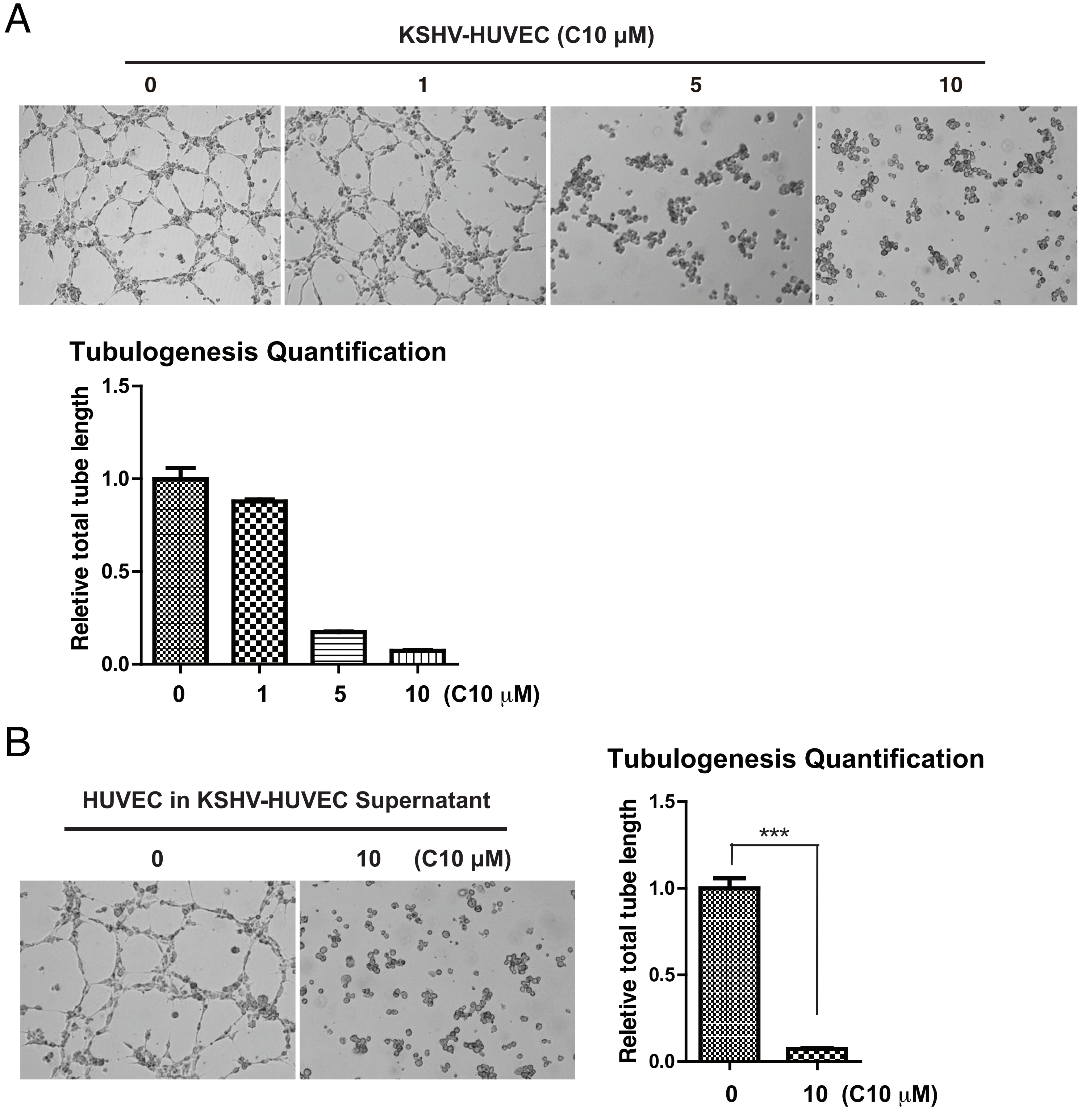

Fig. S2. Effect of C10 on KSHV-mediated paracrine regulation of angiogenesis of KSHV-infected HUVEC. (A) KSHV-infected HUVECs were placed on Matrigel in the presence of C10 in different concentrations. Tubulogenesis was examined under a microscope and quantified by measuring the total tube length using the Image J software. (B) Effects of C10 on tubulogenesis of HUVECs treated with conditioned medium of KSHV-infected HUVECs culture were analyzed on Matrigel.
