# Supplementary material for: An APE1 inhibitor reveals critical roles of the redox function of APE1 in KSHV replication and pathogenic phenotypes
Source: PLoS Pathog. 2017 Apr 5;13(4):e1006289. doi: 10.1371/journal.ppat.1006289 (PMC5381946; doi:10.1371/journal.ppat.1006289)
Supplement: S3 Fig — KSHV-infected HUVECs were treated with either C10 for 24 hours. After changing media to remove C10 from the media, cells were continued to culture for 24 hours. Then the culture media were collected and examined for the expression of VEGF-A, IL-6 and IL-8 by ELISA. (PDF) [file ppat.1006289.s003.pdf]

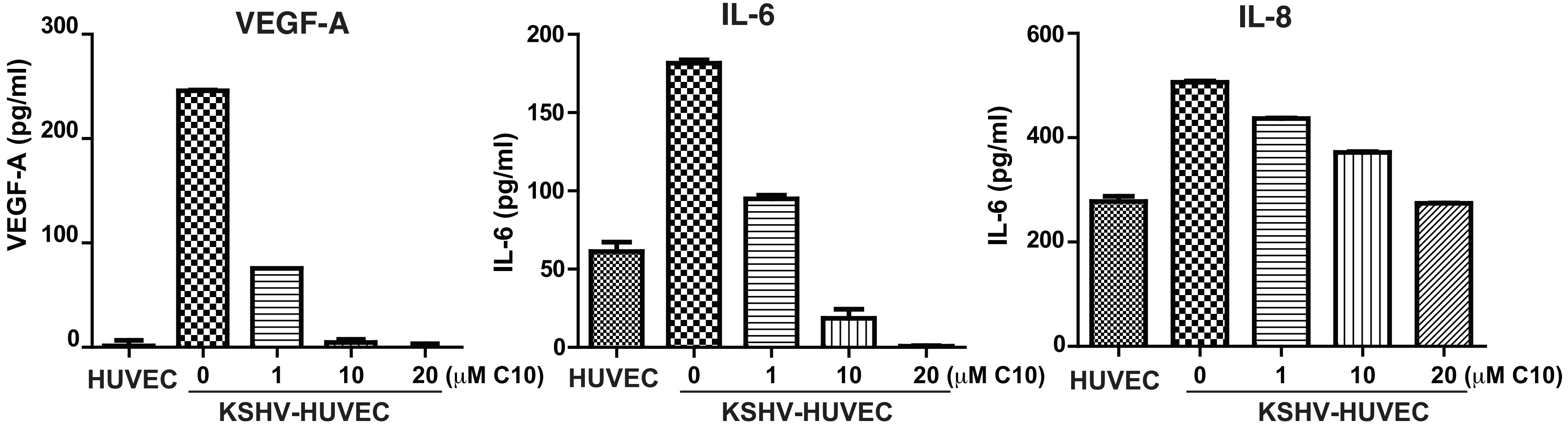

Fig. S3. Effects of C10 on KSHV-mediated secretion of VEGF-A, IL-6 and IL-8 from HUVECs. KSHV-infected HUVECs were treated with either C10 for 24 hours. After changing media to remove C10 from the media, cells were continued to culture for 24 hours. Then the culture media were collected and examined for the expression of VEGF-A, IL-6 and IL-8 by ELISA.
