# Supplementary material for: An APE1 inhibitor reveals critical roles of the redox function of APE1 in KSHV replication and pathogenic phenotypes
Source: PLoS Pathog. 2017 Apr 5;13(4):e1006289. doi: 10.1371/journal.ppat.1006289 (PMC5381946; doi:10.1371/journal.ppat.1006289)
Supplement: S4 Fig — (PDF) [file ppat.1006289.s004.pdf]

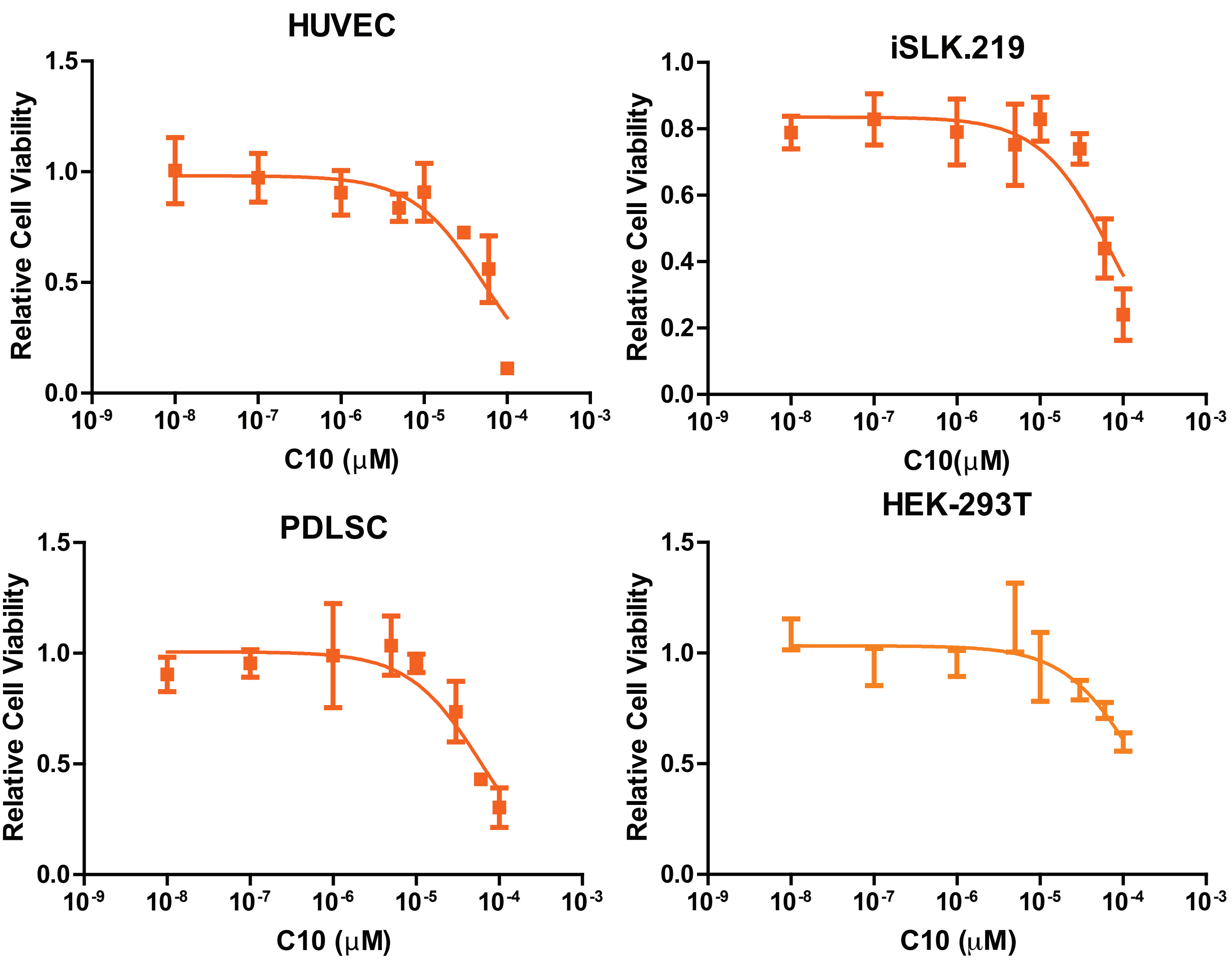

Summary of Cytotoxicity of C10 in Different Types of Cell

Cell	HUVEC	PDLSC	iSL.219	HEK-293T
C10	53 μM	60 μM	75 μM	143 μM

Fig. S4. Cytotoxicities of C10 to HUVEC, PDLSC, iSLK.219 and HEK293T cells were assessed after treatment with C10 of different concentrations as indicated for 48 hours using MTT assay.
