# Supplementary figures and images for: An APE1 inhibitor reveals critical roles of the redox function of APE1 in KSHV replication and pathogenic phenotypes
Source: PLoS Pathog. 2017 Apr 5;13(4):e1006289. doi: 10.1371/journal.ppat.1006289 (PMC5381946; doi:10.1371/journal.ppat.1006289)

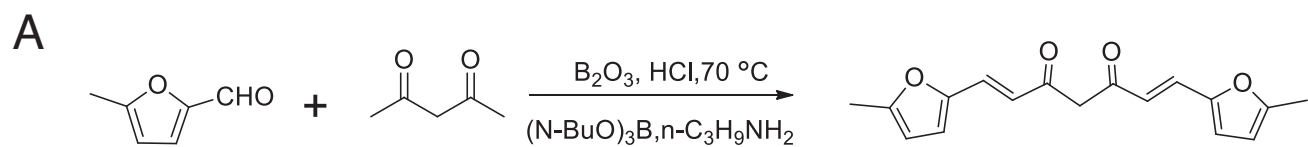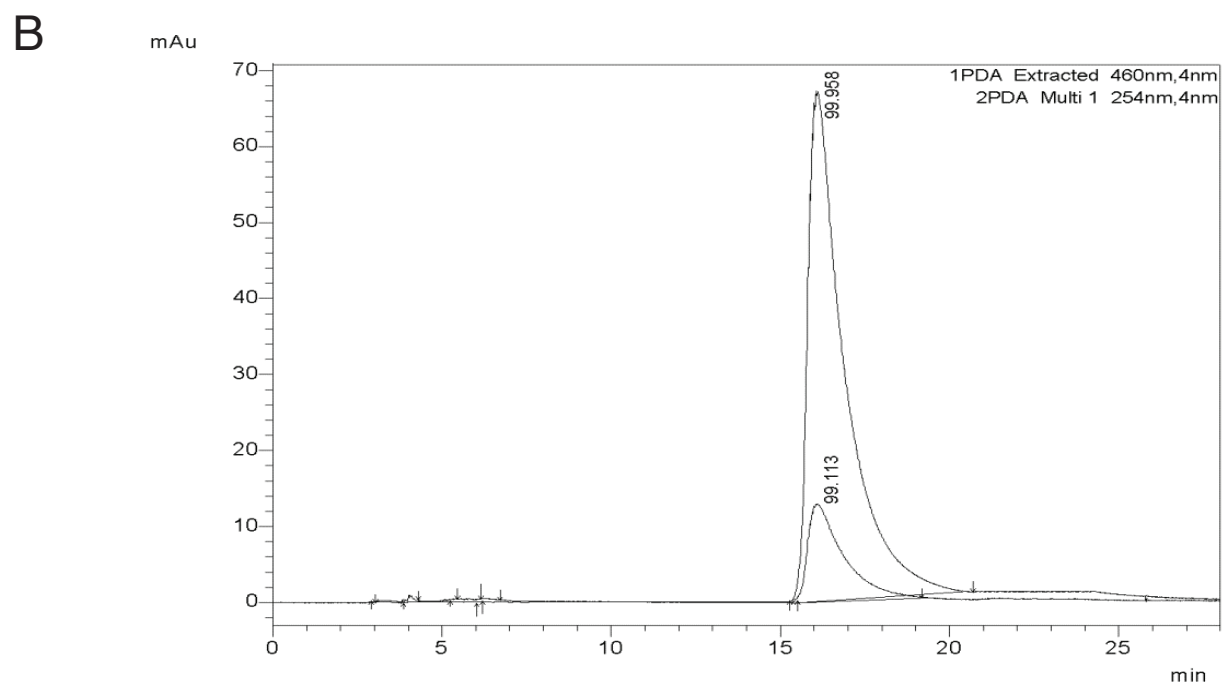

Fig. S5. Synthesis of C10 and HPLC spectrum of the compound.

Supplement: S5 Fig — (PDF) [file ppat.1006289.s005.pdf]

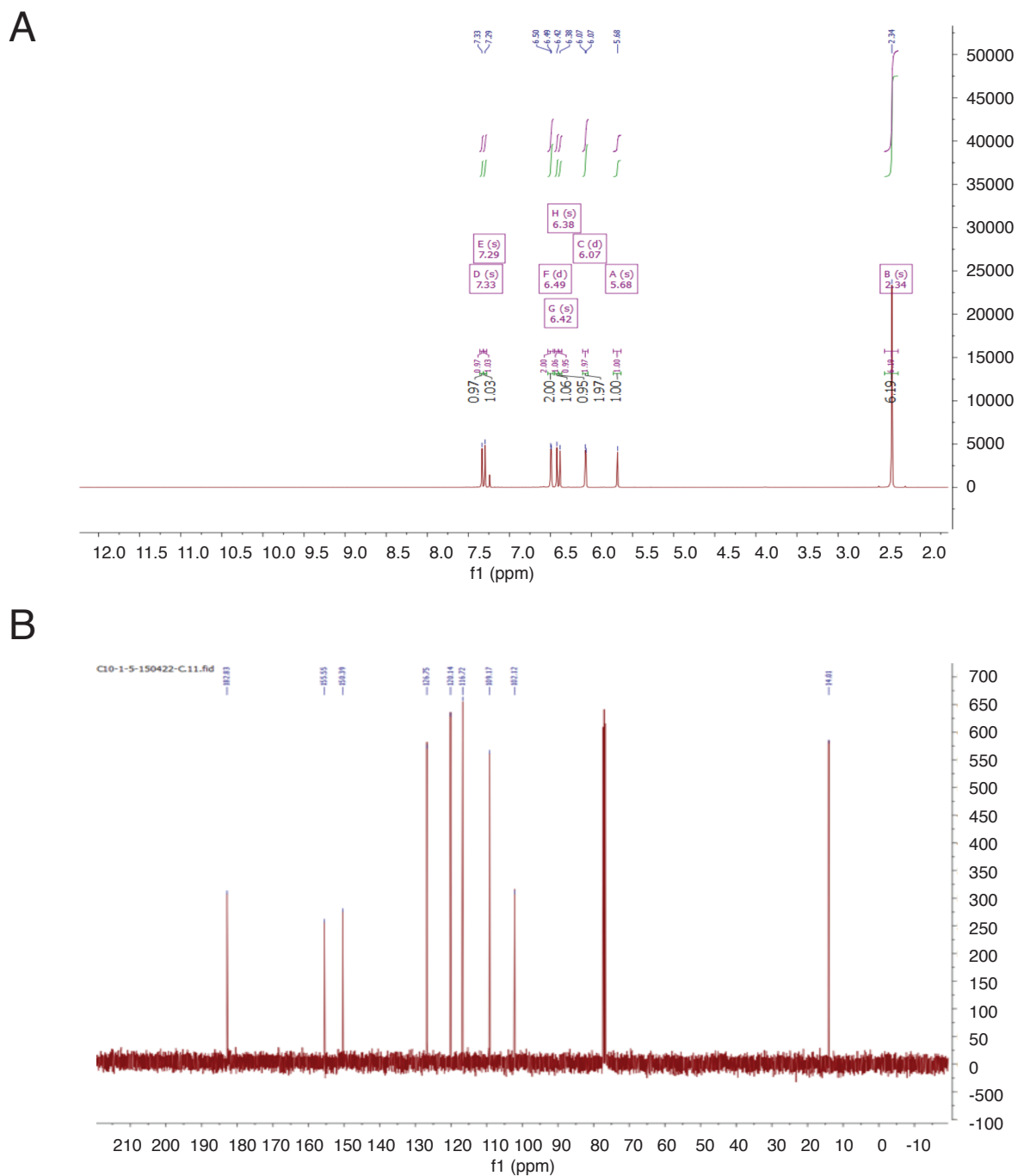

Fig. S6. (A)  $^1\text{H}$ -NMR spectrum of C10 in  $\text{CDCl}_3$  (400MHz) and (B)  $^{13}\text{C}$ -NMR spectrum of C10 in  $\text{CDCl}_3$  (400MHz).

Supplement: S6 Fig — (PDF) [file ppat.1006289.s006.pdf]
