# Supplementary material for: An APE1 inhibitor reveals critical roles of the redox function of APE1 in KSHV replication and pathogenic phenotypes
Source: PLoS Pathog. 2017 Apr 5;13(4):e1006289. doi: 10.1371/journal.ppat.1006289 (PMC5381946; doi:10.1371/journal.ppat.1006289)
Supplement: S7 Fig — (A) His-tagged APE1 was expressed in E. coli, purified by Nickel affinity chromatography with Ni2+-NTA-resin and eluted with buffer containing imidazole. (B) HPLC spectrum of obtained APE1 to show purify of APE1 used in the DSF, CD, SPR and EMSA assays. (PDF) [file ppat.1006289.s007.pdf]

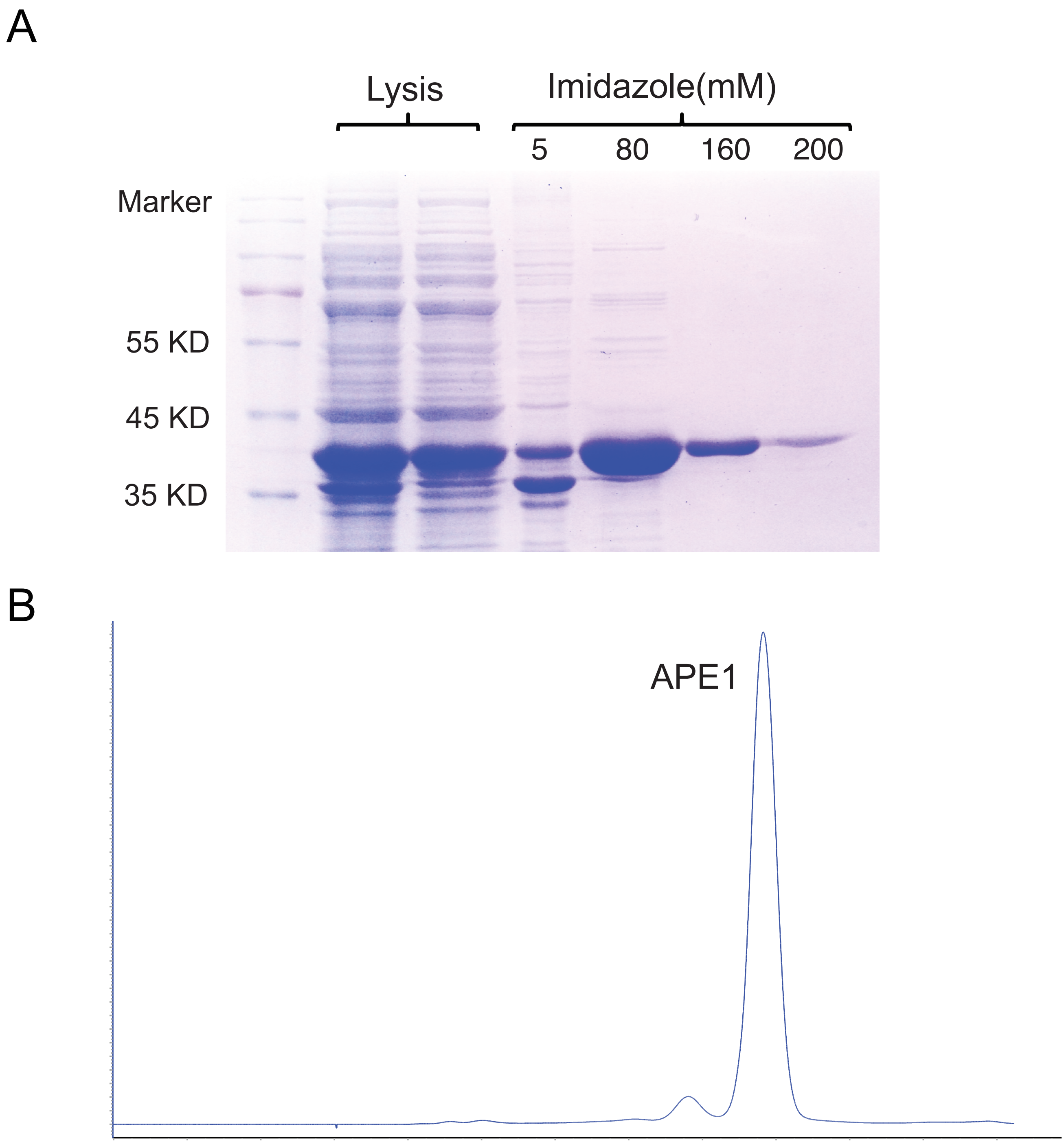

Fig. S7. Purification and purity of APE1 protein used in the study. (A) His-tagged APE1 was expressed in *E. coli*, purified with Ni2+-NTA-resin and eluted with buffer containing imidazole. (B) HPLC spectrum of obtained APE1 to show purify of APE1 used in the DSF, CD, SPR and EMSA assays.
